# Natural Bioactive Compounds from *Delonix regia* Seeds Revealed Through Integrated Phytochemical, Biomedical and In Silico Evaluation

**DOI:** 10.3390/ph19081272

**Published:** 2026-08-12

**Authors:** Husam Qanash, Aisha M. H. Al-Rajhi, Abdulrahman S. Bazaid, Manar F. Alghassab, Fahad Almarshadi, Walid Alesefir, Waleed Hakami, Amro Duhduh, Abdu Aldarhami

**Affiliations:** 1 Department of Medical Laboratory Science, College of Applied Medical Sciences, University of Ha’il, Hail 55476, Saudi Arabia; 2 Medical and Diagnostic Research Center, University of Ha’il, Hail 55473, Saudi Arabia; 3 Department of Biology, College of Science, Princess Nourah bint Abdulrahman University, P.O. Box 84428, Riyadh 11671, Saudi Arabia; 4 College of Dentistry, University of Ha’il, Hail 55473, Saudi Arabia; 5 Public Health Department, College of Public Health and Health Informatics, University of Ha’il, Hail 55473, Saudi Arabia; 6 Department of Internal Medicine, College of Medicine, University of Ha’il, Hail 55473, Saudi Arabia; 7 Department of Medical Laboratory Technology, College of Nursing and Health Sciences, Jazan University, Jazan 45142, Saudi Arabia; 8 Department of Microbiology and Parasitology, Faculty of Medicine, Umm Al-Qura University, Al-Qunfudah 28815, Saudi Arabia

**Keywords:** *Delonix regia*, anticancer activity, *Helicobacter pylori*, antibiofilm activity, wound healing, anti-inflammatory activity, anticoagulant activity

## Abstract

**Background/Objectives:** *Delonix regia* seeds contain phytochemicals with therapeutic potential, but their activity against *Helicobacter pylori* and biomedical properties remain incompletely characterized. This study aimed to characterize the phenolic and amino acid composition of *D. regia* seed extract (DRSE) and evaluate its anticancer, wound-healing, anti-inflammatory, anticoagulant, antibacterial, antibiofilm and urease-targeted docking activities. **Methods:** DRSE was analyzed by high-performance liquid chromatography (HPLC) and amino acid analysis. Biological effects were assessed using MTT cytotoxicity, scratch wound-healing, bovine serum albumin (BSA) denaturation, prothrombin time (PT) and partial thromboplastin time (PTT), antibacterial and crystal violet antibiofilm assays. Gallic acid and vanillin were docked against *H. pylori* urease (PDB ID: 1E9Y). **Results:** Gallic acid was the predominant phenolic compound (1417.90 µg/g), while aspartic and glutamic acids dominated the amino acid fraction. DRSE showed preferential cytotoxicity toward A431 carcinoma cells (IC_50_ = 108.97 ± 0.68 µg/mL) compared with HFB4 fibroblasts (IC_50_ = 318.56 ± 2.06 µg/mL), yielding a selectivity index of 2.92. Scratch closure was comparable to the control (81.71% versus 80.85%), although the migration rate increased to 16.12 µm. DRSE inhibited protein denaturation by 91.20% (IC_50_ = 6.39 ± 0.19 µg/mL) and prolonged PT and PTT to 27.37 and 90.50 s, respectively. It inhibited *H. pylori* with minimum inhibitory and bactericidal concentrations of 31.25 µg/mL and suppressed biofilm formation by 95.39%. Gallic acid and vanillin showed comparable urease docking scores of −4.72 and −4.71 kcal/mol. **Conclusions:** DRSE showed selective anticancer, anti-*H. pylori*, antibiofilm, anti-inflammatory, anticoagulant, and moderate pro-migratory activities. Mechanistic, safety and in vivo studies are warranted.

## 1. Introduction

Natural products remain a major source of structurally diverse molecules for pharmaceutical, nutraceutical and industrial development. Plant-derived metabolites are of particular interest because they display broad biological activity and may provide sustainable alternatives or complements to synthetic agents [1,2]. Their recovery, however, depends strongly on the chemical composition of the plant matrix, solvent polarity, extraction conditions and post-extraction processing [3]. Consequently, substantial effort has been directed toward improving extraction strategies to maximize phytochemical yield and quality while reducing environmental burden [4,5]. Within this context, *Delonix regia* (Poinciana) has emerged as a promising yet incompletely explored source of biologically active metabolites [6].

*Delonix regia* belongs to the Fabaceae family and the Caesalpinioideae subfamily. The species is widely distributed across tropical and subtropical regions of Africa, Asia, and the Americas, reflecting its ecological adaptability and considerable horticultural importance. Beyond its ornamental value, different parts of the plant have been used or investigated as sources of polysaccharides, phenolics, flavonoids, proteins, and other functional constituents [7].

The seeds of *D. regia* have attracted increasing attention because of their diverse phytochemical composition and reported biological activities. Seed extracts have shown antifungal, antibacterial, and antimalarial effects, supporting their potential as a source of natural therapeutic agents [8,9,10]. Krishnaraj et al. [11] further demonstrated that polysaccharides isolated from *D. regia* seeds possess favorable physicochemical properties and low toxicity, indicating potential pharmaceutical utility. Other plant organs also display relevant biological activity. Phytochemical studies of flowers have identified several bioactive flavonoids, and flower extracts have demonstrated antioxidant and antiproliferative effects against multiple cancer cell lines [12]. The bark has likewise shown antimicrobial, wound repair-promoting, anti-plasmodial and free-radical-scavenging activities, underscoring the pharmacological relevance of the species beyond its seeds and flowers [13]. In an in vivo model of renal impairment, dietary supplementation with *D. regia* improved biochemical indices of kidney and liver function, favorably influenced lipid metabolism, and reduced histopathological injury [14].

*Helicobacter pylori* is a persistent gastric pathogen that colonizes the stomach mucosa and contributes to chronic gastritis, peptic ulcer disease, and gastric carcinogenesis [15]. The continuing emergence of antimicrobial resistance and the capacity of *H. pylori* to form biofilms create a need for new agents capable of targeting both planktonic and sessile bacterial populations. At the same time, cancer remains a major global health challenge; GLOBOCAN 2022 estimated approximately 20 million new cases and 9.7 million deaths worldwide [16]. Chronic or impaired wound healing is another complex clinical problem driven by dysregulated inflammation, oxidative stress, infection, and defective tissue repair, with substantial consequences for morbidity and healthcare expenditure [17]. Plant extracts capable of addressing several of these interconnected processes would therefore be scientifically and therapeutically relevant.

Molecular docking provides a complementary computational framework for predicting how phytochemicals may interact with biologically relevant targets and for prioritizing compounds for mechanistic validation [18,19,20]. Integrating in silico analysis with chemical profiling and experimental bioassays can strengthen the interpretation of natural-product studies by linking extract composition to plausible molecular mechanisms [21,22].

Recent studies have increasingly combined in vitro biological assays with molecular docking to strengthen the evaluation of the pharmacological potential of medicinal plants. For example, essential oils derived from *Curcuma heyneana* and *Kaempferia galanga* exhibited notable biological activities, which were further supported by molecular docking analyses [23]. Similarly, docking studies of bioactive compounds isolated from *Stevia rebaudiana* provided mechanistic insights into their antioxidant effects, highlighting the value of integrating computational and experimental approaches in natural-product research [24].

Despite the growing literature on *D. regia*, an integrated evaluation of the seed extract encompassing anti-*H. pylori* and antibiofilm effects, selective anticancer activity, wound healing, inflammation, coagulation, and urease-targeted docking has not been adequately reported. Therefore, the present study aimed to characterize the phenolic and amino acid composition of *D. regia* seed extract (DRSE) and to assess its multifunctional biological activities using complementary in vitro assays. In addition, gallic acid and vanillin were docked against *H. pylori* urease to explore whether both major and minor phytochemical constituents could contribute to the observed antibacterial activity.

## 2. Results and Discussion

### 2.1. Botanical Characteristics and Experimental Workflow for D. regia Seed Extract (DRSE) Preparation

**Figure 1:**
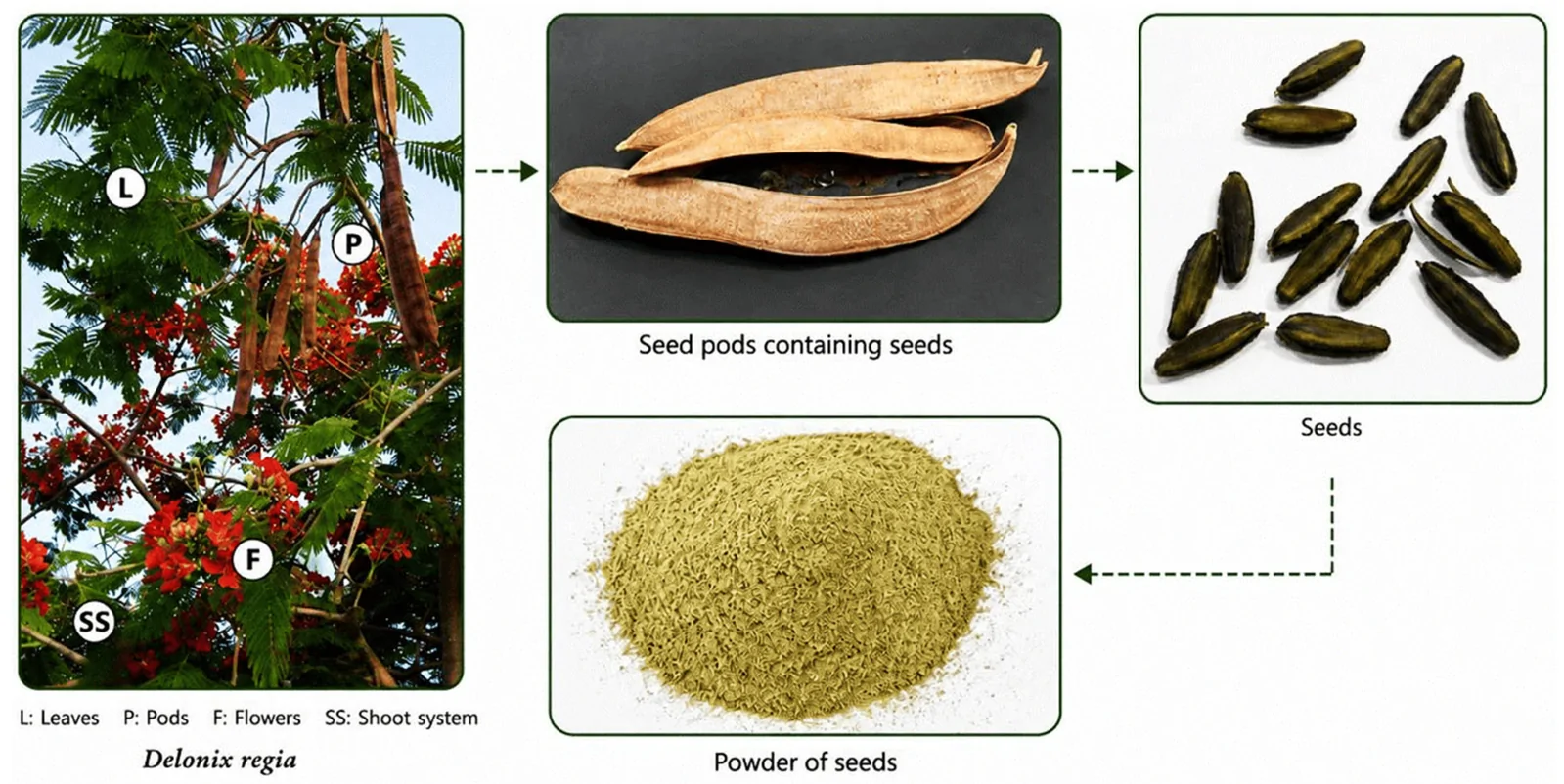
*D. regia* plant material and seed-processing workflow. The shoot system (**SS**) shows leaves (**L**), flowers (**F**) and pods (**P**), followed by seed separation and preparation of the seed powder used for extraction.

The botanical features of *D. regia* and the workflow used to obtain the seed powder are presented (Figure 1). The resulting methanolic seed extract was subjected to chemical characterization and a series of complementary biological assays. The findings are presented below in an integrated manner to relate the phytochemical composition of *D. regia* seed extract (DRSE) to its observed biological effects.

### 2.2. Phenolic and Amino Acid Profiling Reveals the Chemical Richness of D. regia Seed Extract (DRSE)

The phenolic and amino acid profiles of DRSE are summarized (Table 1). Gallic acid was the dominant phenolic constituent (1417.90 µg/g), followed by naringenin (333.18 µg/g), rosmarinic acid (284.01 µg/g), chlorogenic acid (263.22 µg/g), quercetin (247.63 µg/g), catechin (237.95 µg/g), and ellagic acid (207.36 µg/g). Lower concentrations of ferulic acid, rutin, p-coumaric acid, daidzein, syringic acid, and vanillin were also detected. Methyl gallate, caffeic acid, cinnamic acid, kaempferol, and hesperetin were not detected under the chromatographic conditions used, suggesting that these compounds were either absent from the extract or present at concentrations below the method’s limit of detection. This profile identifies DRSE as a phenolic-rich extract dominated by gallic acid but containing a chemically diverse mixture of flavonoids and phenolic acids.

**Figure 2:**
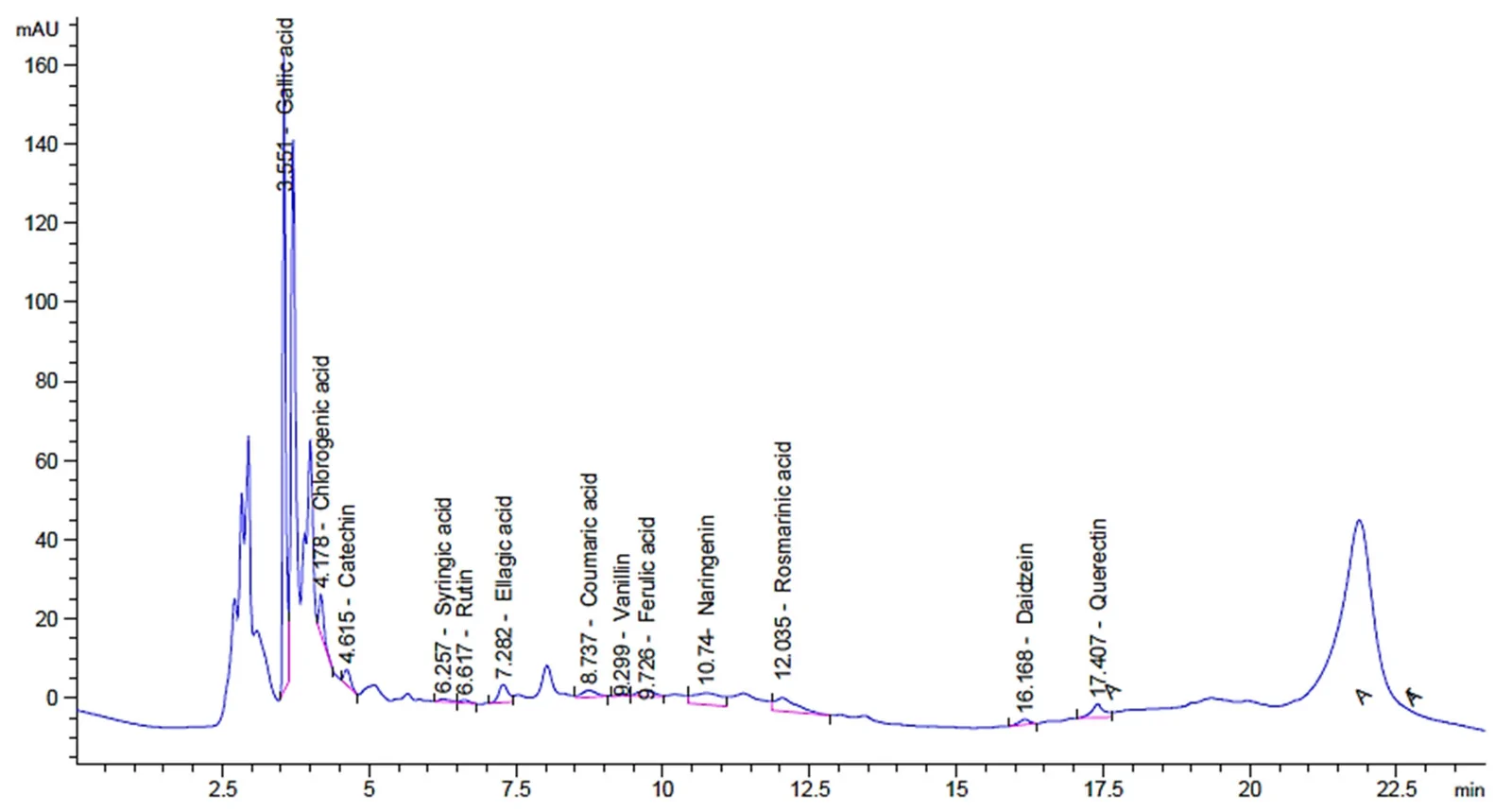
HPLC showing the phenolic profile of *D. regia* seed extract.

HPLC analysis confirmed the chemical diversity of DRSE, with 15 phenolic compounds identified under the applied chromatographic conditions (Figure 2). Previous investigations of *D. regia* leaves, flowers, and bark have similarly identified gallic, protocatechuic, chlorogenic, salicylic, and trans-cinnamic acids, together with other hydroxycinnamic acid derivatives [25]. Although phytochemical composition varies with plant organ, geography, maturity, solvent, and extraction conditions, the present findings are consistent with *D. regia* being a rich source of phenolic metabolites. The high abundance of gallic acid and the presence of naringenin, rosmarinic acid, chlorogenic acid, quercetin, catechin, and ellagic acid provide a plausible chemical basis for the broad biological activity observed in this study. These compounds have been associated with antimicrobial, antibiofilm, antioxidant, anti-inflammatory, and tissue-protective effects. Accordingly, the DRSE profile complements previous reports describing antioxidant, antimicrobial, hepatoprotective, and anti-inflammatory properties across different parts of *D. regia* [26]. Taken together, these findings establish DRSE as a chemically diverse, gallic acid-rich phytochemical reservoir whose composite phenolic profile may underpin its broad biological activities and support its further development as a promising source of natural bioactive agents.

**Figure 3:**
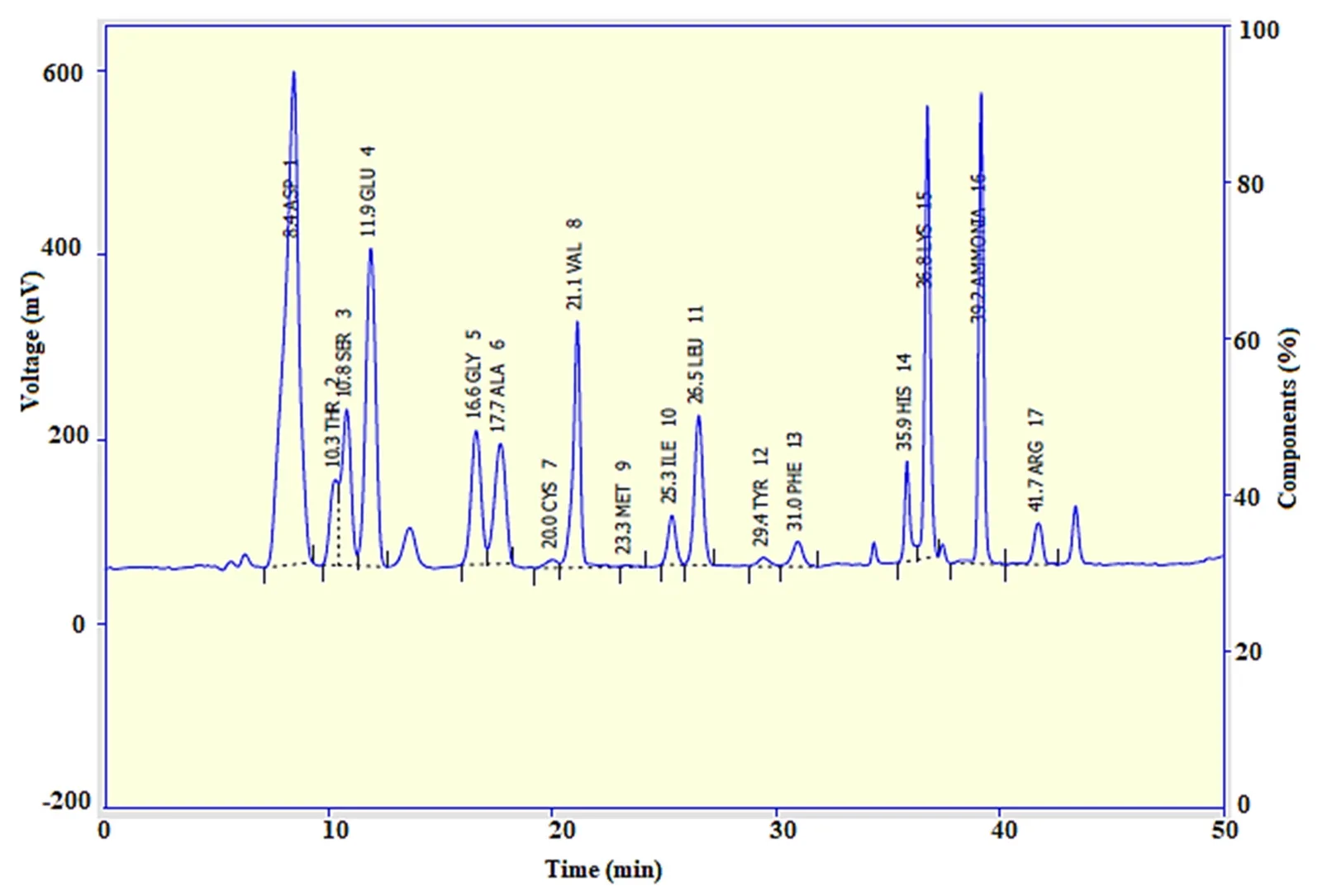
Amino acid chromatogram of *D. regia* seed extract.

The chromatogram of 18 amino acids is reported (Figure 3). Aspartic acid was the predominant amino acid (274.68 mg/g), followed by glutamic acid (109.88 mg/g). Glycine, ammonia, leucine, valine, serine, threonine, lysine, and proline were present at intermediate concentrations, whereas cysteine and methionine occurred at comparatively low levels. This predominance of acidic amino acids is broadly consistent with previous analyses of plant-derived protein materials and may contribute to the nutritional and functional properties of the seeds [27]. *D. regia* seeds also contain proteins, lectins, fatty acids, free amino acids, galactomannan, minerals, and vitamins [28,29]. Earlier work reported glutamic acid as the predominant non-essential amino acid in *D. regia* seeds, with leucine as the most abundant essential amino acid [30]. Although the absolute concentrations differ from those observed here, likely because of differences in sample preparation and analytical expression, both studies support the view that *D. regia* seeds possess a substantial and compositionally diverse amino acid fraction. Collectively, this distinctive amino acid profile highlights DRSE as a nutritionally and functionally valuable seed extract, with its abundance of acidic and essential amino acids further supporting the therapeutic and biotechnological potential of *D. regia* seeds.

### 2.3. D. regia Seed Extract (DRSE) Exhibits Preferential Cytotoxicity Toward A431 Carcinoma Cells

**Figure 4:**
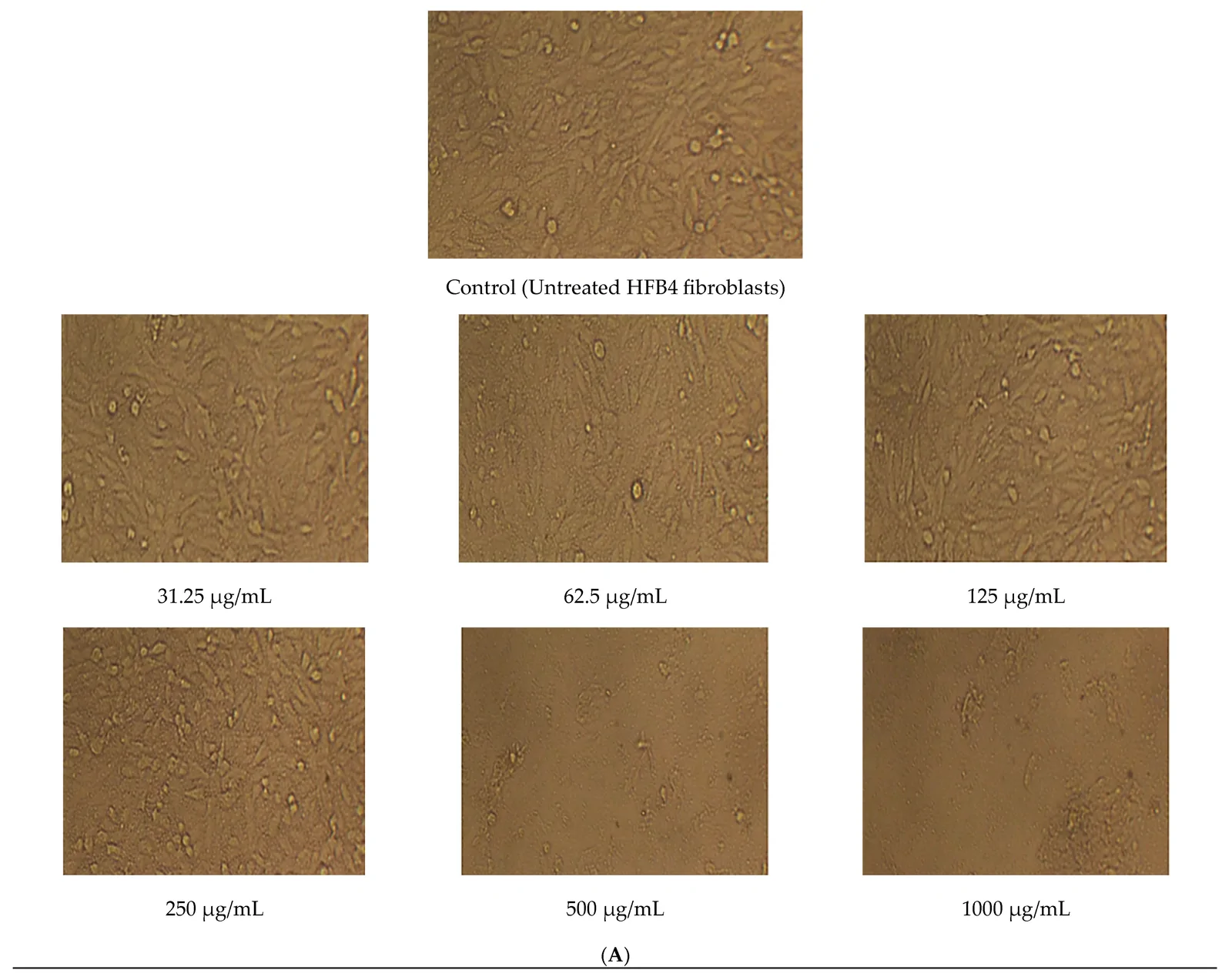

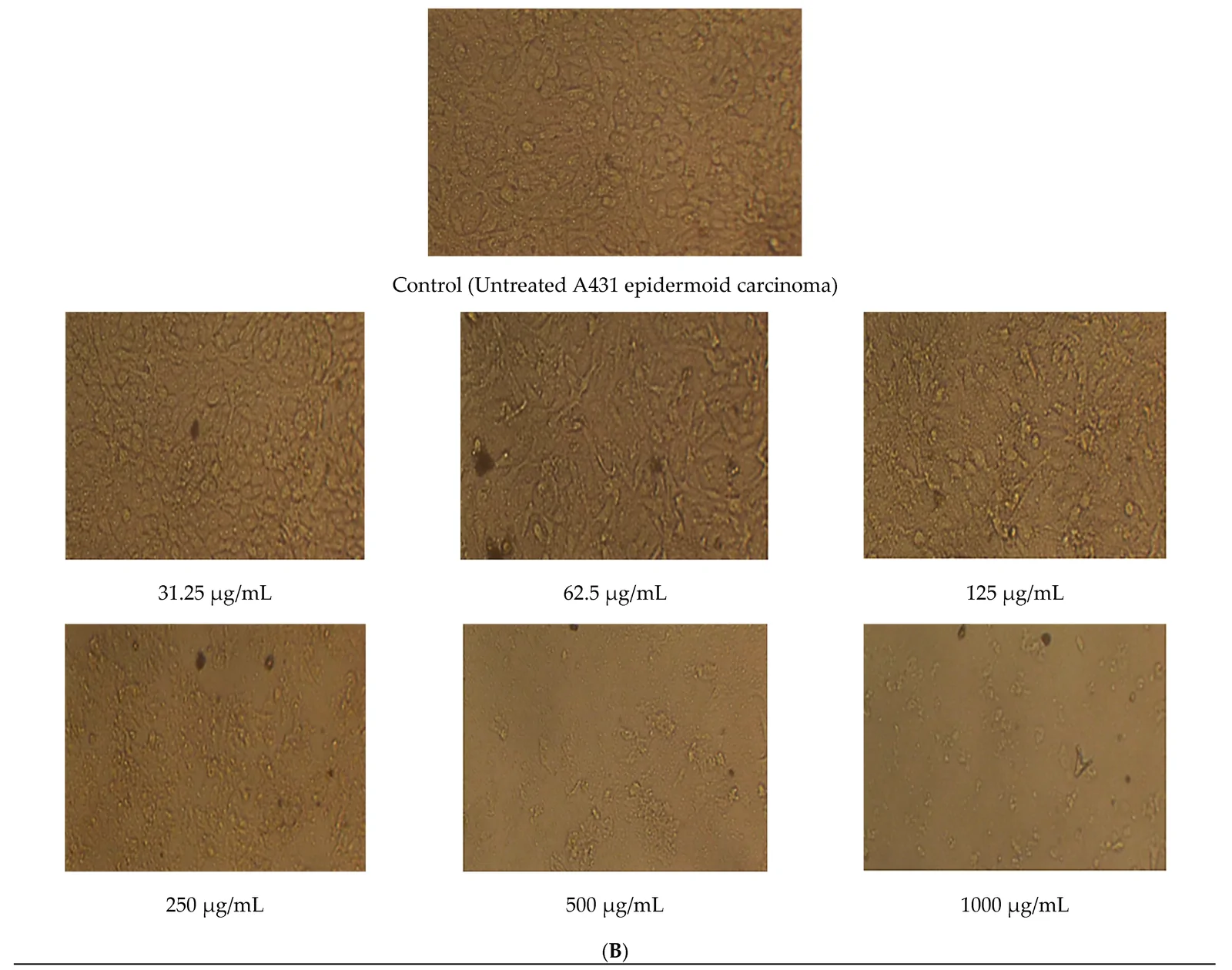
Concentration-dependent morphological changes in (**A**) HFB4 fibroblasts and (**B**) A431 epidermoid carcinoma cells after treatment with *D. regia* seed extract. Scale bar = 100 µm.

**Figure 5:**
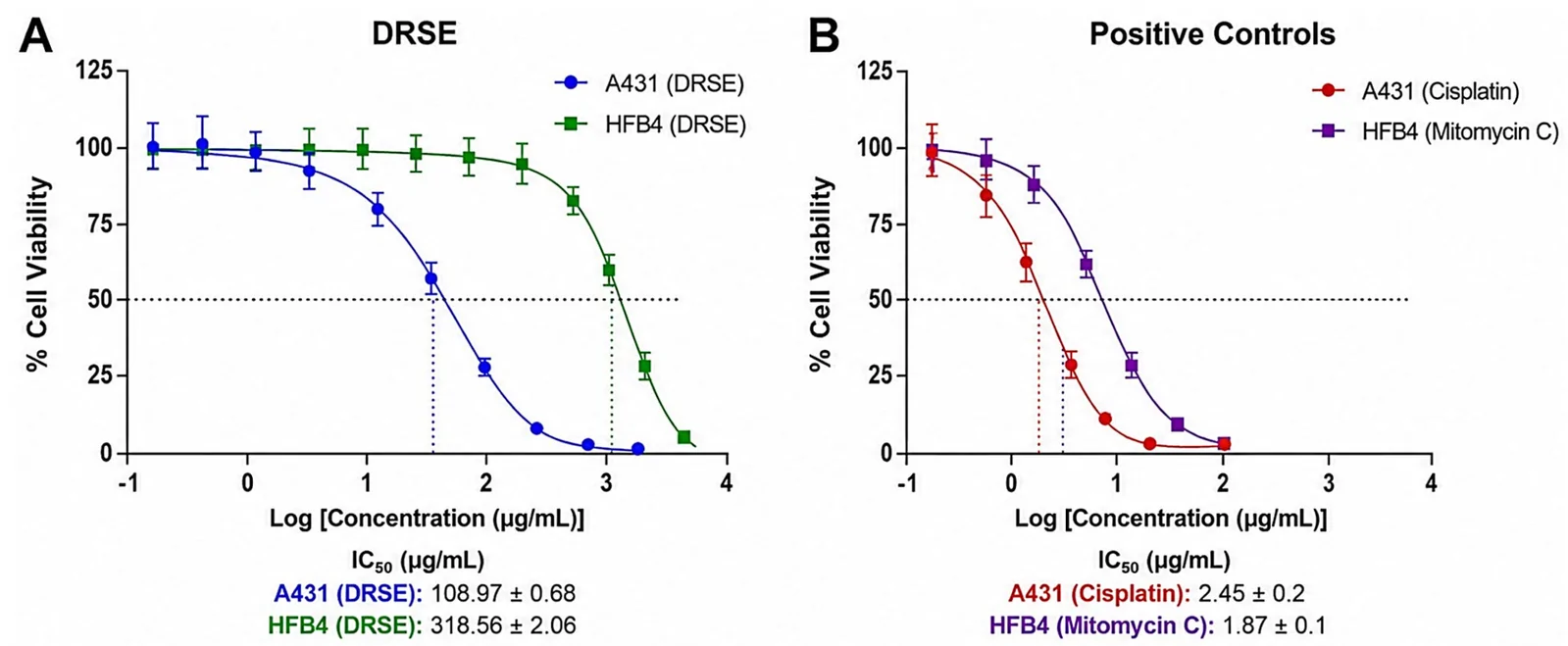
Dose–response curves showing (**A**) the effects of *D. regia* seed extract (DRSE) on A431 and HFB4 cells, together with those of the corresponding reference compounds, (**B**) cisplatin for A431 cells and mitomycin C for HFB4 cells. Cells were treated for 48 h, and cell viability was assessed using the MTT assay. Data are presented as the mean ± standard deviation of three independent experiments, each performed in triplicate. IC_50_ values were determined by nonlinear regression analysis using GraphPad Prism version 8.0. DRSE exhibited greater cytotoxicity toward A431 cells (IC_50_ = 108.97 µg/mL) than toward HFB4 cells (IC_50_ = 318.56 µg/mL). Cisplatin and mitomycin C produced IC_50_ values of 2.45 µM and 1.87 µg/mL, respectively, and served as positive controls for the assay.

The cytotoxic effects of DRSE on HFB4 fibroblasts and A431 epidermoid carcinoma cells are reported (Table 2 and Figure 4). DRSE produced a clear concentration-dependent response, with A431 cells showing substantially greater sensitivity than HFB4 cells. At 62.5 and 125 µg/mL, cytotoxicity in A431 cells reached 30.23 ± 0.15% and 55.23 ± 0.04%, respectively, whereas HFB4 cytotoxicity remained 0.11 ± 0.01% and 2.42 ± 0.03%. At 250 µg/mL, cytotoxicity increased to 85.75 ± 0.89% in A431 cells but remained 30.07 ± 0.05% in HFB4 cells. The IC_50_ values were 108.97 ± 0.68 µg/mL for A431 cells and 318.56 ± 2.06 µg/mL for HFB4 cells, yielding an approximate selectivity index of 2.92. IC_50_ values were determined by nonlinear regression analysis of dose–response curves generated from three independent experiments; each was performed in triplicate. Cisplatin (10 µM) and mitomycin C (5 µg/mL) were used as positive controls for A431 and HFB4 cells, respectively. The dose–response curves for DRSE and the corresponding reference compounds are documented (Figure 5). The relatively small standard errors indicate good reproducibility across the independent experiments conducted under standardized assay conditions. These data indicate preferential activity against the malignant cell line within the tested concentration range. The selectivity may reflect the combined action of phenolic acids and flavonoids capable of modulating oxidative balance, cell-cycle progression, mitochondrial function, and apoptotic signaling. Comparable antiproliferative effects have been reported for extracts from *D. regia* flowers and leaves across liver, breast, brain, cervical, colon, and other cancer cell models [12,31,32]. Nevertheless, because the present assay measured metabolic viability rather than a specific death pathway, apoptosis and cell-cycle mechanisms should be confirmed experimentally before definitive mechanistic conclusions are drawn.

Phase-contrast micrographs corroborated the quantitative viability data (Figure 4). Untreated HFB4 and A431 cells retained their characteristic morphology, attachment, and confluence. Increasing DRSE concentrations caused progressive loss of cell density, cell rounding, shrinkage, membrane irregularity, monolayer disruption, and detachment. These changes were more pronounced in A431 cells, particularly at intermediate concentrations, whereas extensive damage was evident in both cell lines at 500 and 1000 µg/mL. The morphological findings therefore support a concentration-dependent cytotoxic effect and greater susceptibility of A431 carcinoma cells to DRSE. Taken together, the marked differential sensitivity of A431 and HFB4 cells, supported by both viability and morphological evidence, highlights DRSE as a promising source of selectively acting anticancer constituents and warrants further mechanistic and in vivo investigation. The positive controls, cisplatin (10 µM) for A431 cells and mitomycin C (5 µg/mL) for HFB4 cells, produced the expected cytotoxic effects, supporting the validity and reliability of the assay. The dose-dependent cytotoxic responses of DRSE and the corresponding reference compounds are reported (Figure 5).

The cytotoxic activity of DRSE is consistent with the well-established anticancer properties of gallic acid, which was identified as the predominant phenolic constituent of the extract. In the present study, pure gallic acid exhibited cytotoxic activity against A431 cells, with an IC_50_ value of 178.25 ± 0.66 µg/mL. However, DRSE showed significantly greater potency, with an IC_50_ value of 108.97 ± 0.68 µg/mL, indicating that the crude extract was approximately 1.64-fold more active than gallic acid alone. Gallic acid also displayed low toxicity toward normal HFB4 cells, with an IC_50_ value exceeding 400 µg/mL, whereas DRSE produced an IC_50_ value of 318.56 ± 2.06 µg/mL and a selectivity index of approximately 2.92. Gallic acid has been widely reported to inhibit cancer cell growth through several mechanisms, including the induction of apoptosis, generation of reactive oxygen species, cell-cycle arrest, suppression of oncogenes and matrix metalloproteinases, and modulation of the phosphoinositide 3-kinase/protein kinase B, nuclear factor kappa B, and mitogen-activated protein kinase signaling pathways [33,34,35]. These effects have been demonstrated in several cancer models, including hepatocellular carcinoma [33], breast cancer [35], and other human malignancies [34]. Nevertheless, the greater cytotoxic potency of DRSE compared with pure gallic acid suggests that the anticancer activity of the extract cannot be attributed to gallic acid alone. Rather, the enhanced effect may reflect additive or synergistic interactions between gallic acid and other bioactive constituents detected in DRSE, including naringenin, rosmarinic acid, chlorogenic acid, quercetin, catechin, and ellagic acid. Such interactions may enhance the biological activity of botanical extracts by modulating multiple cellular targets and signaling pathways simultaneously.

### 2.4. Pro-Migratory and Wound-Healing Effects of D. regia Seed Extract (DRSE)

**Figure 6:**
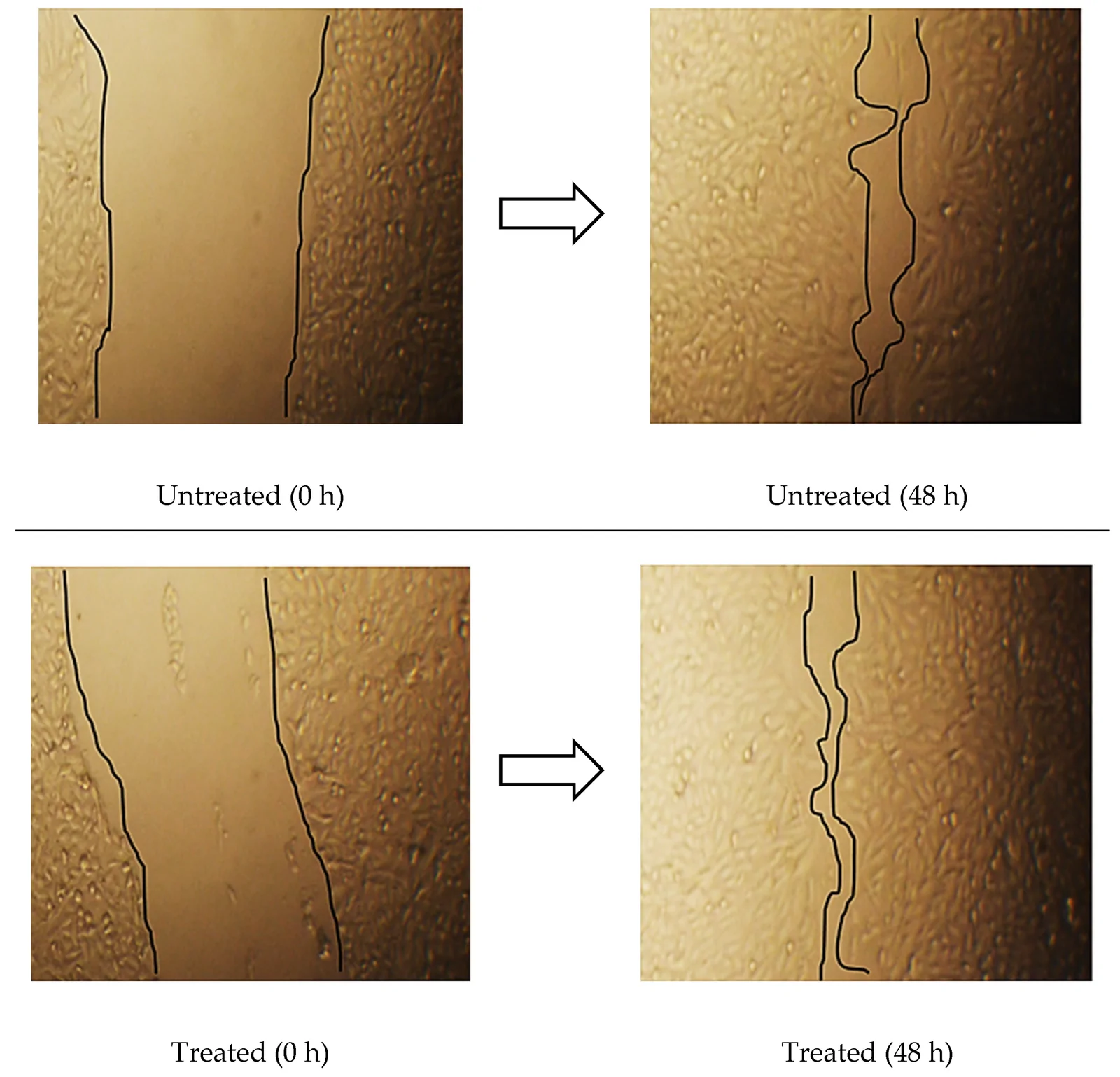
Representative scratch-assay images of untreated and *D. regia* seed extract-treated HFB4 fibroblasts at 0 and 48 h. Scale bar = 200 µm.

Wound-healing potential of DRSE by comparing wound closure parameters with the untreated control after 48 h was demonstrated (Table 3 and Figure 6). DRSE was evaluated at a concentration of 50 µg/mL, selected on the basis of preliminary cytotoxicity data demonstrating greater than 90% viability in HFB4 cells at this concentration. At the initial time point (0 h), the scratch width and wound area were 844.67 µm and 908,618.41 µm^2^ in the control group, whereas the DRSE-treated cells exhibited slightly larger initial values (946.68 µm and 1,018,351.46 µm^2^). Despite the larger initial wound area, cells treated with DRSE showed a marked reduction in wound dimensions after 48 h, reaching a mean width of 173.07 µm and a mean area of 186,214.1 µm^2^, compared with 161.70 µm and 174,018.5 µm^2^ in the control. The percentage of wound closure was high in both groups, amounting to 80.85% in the control and 81.71% in the DRSE-treated cells. DRSE did not significantly enhance the overall percentage of wound closure compared with the untreated control. Nevertheless, the rate of migration (RM) was significantly higher in the DRSE group (16.12 µm) than in the control (14.23 µm). Similarly, the area difference, representing the extent of wound reduction, was significantly greater in DRSE-treated cells (832,137.4 µm^2^) than in the control (734,599.9 µm^2^). The wound-healing potential of DRSE is in accordance with previous studies on *D. regia* seed constituents, which demonstrated their ability to regulate inflammation and oxidative stress and to enhance growth factor-mediated tissue regeneration [36,37]. These mechanisms may explain the increased migration rate and greater wound area reduction observed in the present study, suggesting that DRSE facilitates the repair process by promoting cellular proliferation and tissue remodeling. After 48 h, wound closure was high in both the untreated control (80.85%) and DRSE-treated cells (81.71%), and the difference in closure percentage was not statistically significant. However, DRSE significantly increased the migration rate from 14.23 to 16.12 µm and increased the absolute wound-area reduction from 734,599.9 to 832,137.4 µm^2^. Because the initial scratch area was larger in the DRSE-treated group, interpretation of the wound-healing results should focus primarily on the migration rate and absolute reduction in scratch area rather than on the nearly identical percentages of wound closure. Overall, the findings suggest that DRSE supported fibroblast migration under experimental conditions but did not markedly improve relative wound closure over the untreated control. This moderate pro-migratory effect is consistent with reports that *D. regia* seed galactomannan can regulate inflammation and oxidative stress and promote growth factor-associated tissue repair [36,37]. Collectively, these findings indicate that DRSE exerts a moderate pro-migratory effect on fibroblasts, as reflected by the increased migration rate and wound-area reduction, while its influence on overall wound closure remains limited and warrants further mechanism and in vivo validation. Several limitations of the scratch assay should be acknowledged. A proliferation inhibitor, such as mitomycin C or hydroxyurea, was not used to suppress cell division during the assay period. Consequently, the observed wound closure likely reflects the combined effects of cell migration and proliferation rather than migration alone. The findings should therefore be interpreted as evidence of the overall wound-healing potential of DRSE, rather than as a specific measure of migratory activity. In addition, the initial scratch areas were not completely uniform across the experimental groups, which is a recognized technical limitation of manually performed scratch assays. Nevertheless, the greater wound-closure rate and absolute reduction in scratch area observed in the DRSE-treated group support a genuine biological effect. Future studies incorporating real-time live-cell imaging, proliferation inhibitors, or dedicated migration assays, such as Boyden chamber or transwell assays, are warranted to distinguish more clearly between the relative contributions of cell migration and proliferation to DRSE-mediated wound repair.

### 2.5. D. regia Seed Extract (DRSE) Inhibits Protein Denaturation in a Concentration-Dependent Manner

**Figure 7:**
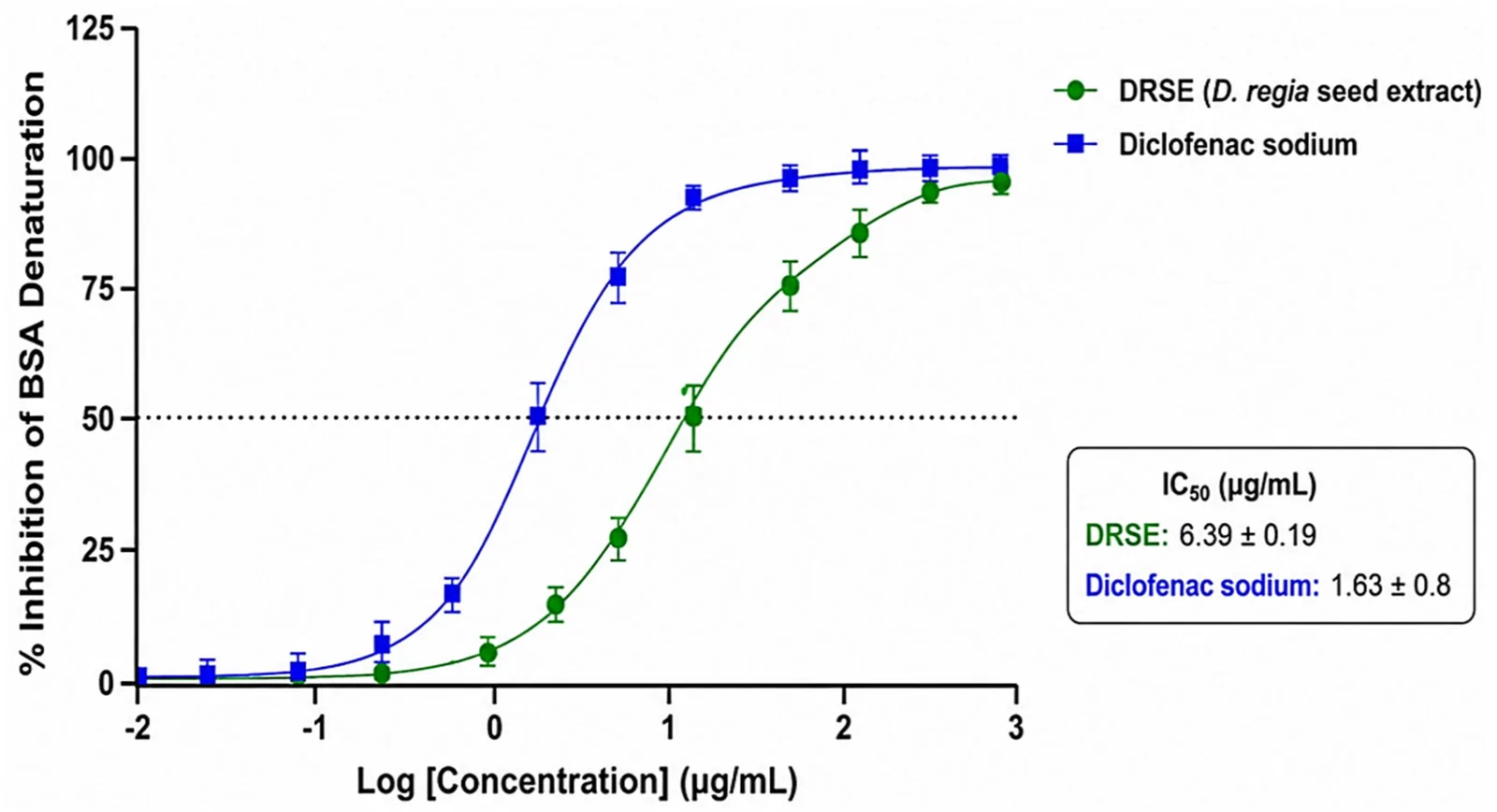
Dose–response curves for the inhibition of bovine serum albumin (BSA) denaturation by *D. regia* seed extract (DRSE) and the reference anti-inflammatory drug diclofenac sodium. Data are presented as the mean ± standard deviation of three independent experiments, each performed in triplicate.

Anti-inflammatory activity of DRSE was evaluated and compared with the standard anti-inflammatory drug diclofenac sodium over a concentration range of 1.56–200 µg/mL (Table 4 and Figure 7). Both samples exhibited a concentration-dependent increase in anti-inflammatory activity. Diclofenac sodium demonstrated significantly higher inhibitory activity than DRSE at all tested concentrations (*p* < 0.05). At the lowest tested concentration (1.56 µg/mL), diclofenac sodium exhibited an inhibition of 47.00 ± 0.87%, whereas DRSE showed 32.50 ± 0.50%. The anti-inflammatory activity progressively increased with increasing concentration, reaching 98.30 ± 0.89% for diclofenac sodium and 91.20 ± 0.26% for DRSE at 200 µg/mL. The dose-dependent response of DRSE indicates the presence of bioactive phytochemicals capable of stabilizing proteins and reducing inflammatory responses, although its activity remained significantly lower than that of the reference drug at all concentrations. Nevertheless, DRSE achieved more than 90% inhibition at the highest tested concentration, suggesting a potent anti-inflammatory potential. The calculated IC_50_ values further confirmed the superior potency of diclofenac sodium. Diclofenac sodium exhibited an IC_50_ value of 1.63 ± 0.80 µg/mL, whereas DRSE showed an IC_50_ of 6.39 ± 0.19 µg/mL, indicating that approximately four times higher concentration of DRSE was required to achieve 50% inhibition. Despite this lower potency relative to the standard drug, the strong concentration-dependent inhibition exhibited by DRSE highlights its potential as a promising natural anti-inflammatory agent. The biological activities observed in the present study are consistent with previous findings reported by Khongkaew et al. [12], who identified rutin, isoquercitrin, and myricetin as the major flavonoids in *D. regia* flower extract. The authors demonstrated remarkable antioxidant activity. These phytochemicals are also recognized for their anti-inflammatory properties, primarily through scavenging reactive oxygen species, suppressing the production of pro-inflammatory mediators, and stabilizing proteins against denaturation. Therefore, the significant concentration-dependent inhibition of bovine serum albumin (BSA) denaturation observed for DRSE in the present study is likely attributable, at least in part, to its flavonoid constituents. The close relationship between antioxidant and anti-inflammatory activities further supports the potential of DRSE as a natural source of multifunctional bioactive compounds with therapeutic value. The anti-inflammatory activity exhibited by DRSE in the present study may be attributed to its high content of phenolic constituents and flavonoids. These compounds have been extensively reported to regulate inflammatory signaling pathways by suppressing the synthesis of pro-inflammatory cytokines and prostaglandins, while simultaneously limiting the generation of reactive oxygen and nitrogen species. For instance, quercetin derivatives and apigenin-related compounds have been shown to inhibit cyclooxygenase (COX) enzymes and protect proteins from denaturation, thereby reducing inflammatory responses. Consequently, the concentration-dependent inhibition of bovine serum albumin denaturation observed in the current investigation is consistent with the established pharmacological properties of *D. regia* phytochemicals and further supports the potential application of DRSE as a natural anti-inflammatory agent [26].

DRSE inhibited heat-induced bovine serum albumin denaturation in a concentration-dependent manner (Table 4). Inhibition increased from 32.50 ± 0.50% at 1.56 µg/mL to 91.20 ± 0.26% at 200 µg/mL. Diclofenac sodium was significantly more active at every tested concentration, reaching 98.30 ± 0.89% inhibition at 200 µg/mL. The corresponding IC_50_ values were 6.39 ± 0.19 µg/mL for DRSE and 1.63 ± 0.80 µg/mL for diclofenac sodium. Thus, although DRSE was approximately fourfold less potent than the reference drug, it produced strong concentration-dependent protection against protein denaturation. The activity may be related to the phenolic and flavonoid content of the extract, particularly compounds capable of scavenging reactive species, stabilizing proteins, and modulating inflammatory mediators. This interpretation is consistent with previous reports of antioxidants and anti-inflammatory flavonoids in *D. regia*, including rutin and related compounds [12,26]. Because protein denaturation is a simplified chemical model of inflammation, confirmation in cellular systems that measure cytokines, cyclooxygenase activity, or redox signaling will be necessary. This pronounced inhibition of protein denaturation reveals the considerable anti-inflammatory potential of DRSE and provides a strong rationale for validating its bioactivity through mechanistic cellular assays and in vivo inflammatory models.

### 2.6. Concentration-Dependent Anticoagulant Activity of D. regia Seed Extract (DRSE) with Preferential Effects on the Intrinsic Pathway

Abnormal intravascular clot formation is a critical event in the development of thrombotic and cardiovascular disorders. Although several synthetic anticoagulants are currently available for the prevention and treatment of thrombosis, their clinical use is often limited by adverse effects, creating a growing interest in identifying plant-derived compounds with effective anticoagulant properties [38]. The anticoagulant activity of DRSE was assessed by determining prothrombin time (PT) and partial thromboplastin time (PTT), employing heparin as the reference anticoagulant (Table 5). DRSE exhibited a concentration-dependent anticoagulant effect, as evidenced by the progressive prolongation of both PT and PTT with increasing extract concentration. PT increased from 12.17 ± 0.29 s at 25 µg/mL to 27.37 ± 0.15 s at 75 µg/mL, whereas PTT increased more markedly from 23.50 ± 0.50 s to 90.50 ± 0.50 s over the same concentration range. Nevertheless, heparin produced a significantly greater anticoagulant response (*p* < 0.05), extending PT to 210.67 ± 0.58 s and PTT to 324.00 ± 1.00 s at 75 µg/mL. The more pronounced prolongation of PTT than PT suggests that DRSE exerts a stronger influence on the intrinsic coagulation pathway while also affecting the common coagulation cascade. These findings indicate that the extract possesses moderate anticoagulant activity, although its efficacy remains lower than that of the standard anticoagulant heparin. The observed anticoagulant effect is likely associated with the phenolic and flavonoid constituents identified in DRSE, which have been reported to interfere with coagulation factor activation and reduce thrombus formation. The present findings are consistent with those of Rahman et al. [39], who demonstrated that the methanolic flower extract of *D. regia* significantly prolonged coagulation time, increasing PT to approximately 41 s and PTT to 50 s at 1 mg/mL, in addition to exhibiting 35.5% clot lysis with negligible hemolytic activity. Although the present study investigated the seed extract, the comparable anticoagulant response suggests that bioactive phytochemicals are distributed throughout different parts of *D. regia*. Similar anticoagulant activities have also been reported for other medicinal plants, including *Thevetia peruviana* [40] and *Cupressus sempervirens* [5], supporting the potential of plant-derived polyphenols as natural anticoagulant agents.

DRSE produced a concentration-dependent prolongation of both PT and PTT (Table 5). At 75 µg/mL, PT increased to 27.37 ± 0.15 s and PTT to 90.50 ± 0.50 s. Heparin produced a much greater effect at the same concentration, extending PT and PTT to 210.67 ± 0.58 and 324.00 ± 1.00 s, respectively. The disproportionately greater increase in PTT suggests that DRSE may affect components of the intrinsic and common coagulation pathways more strongly than the extrinsic pathway. This interpretation remains provisional because the assay does not identify the specific coagulation factor or interaction involved. Phenolic and flavonoid constituents may contribute by altering coagulation enzyme activity, platelet-related processes, or protein interactions. The findings are broadly consistent with the anticoagulant activity previously reported for *D. regia* flower extract and for other polyphenol-rich medicinal plants [5,39,40]. These results highlight DRSE as a potential source of naturally derived anticoagulant constituents, with its preferential prolongation of PTT suggesting a stronger influence on the intrinsic coagulation pathway that merits further factor-specific and in vivo investigation.

### 2.7. D. regia Seed Extract (DRSE) Exhibits Concentration-Dependent Anti-Helicobacter pylori and Antibiofilm Activities

**Figure 8:**
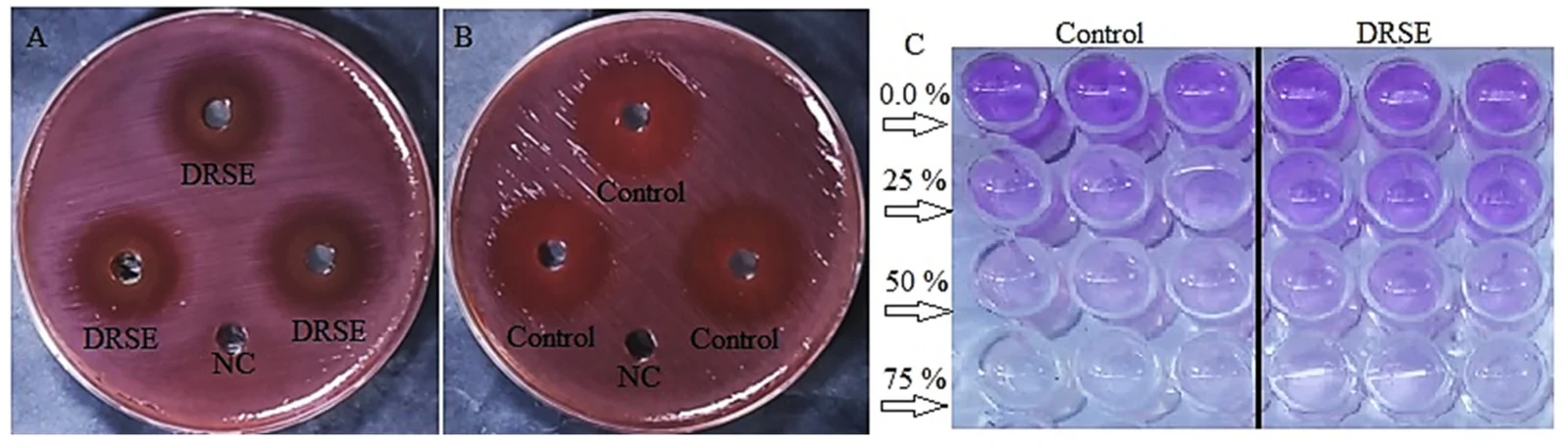
Anti-*H. pylori* activity of *D. regia* seed extract (DRSE). (**A**), Agar-well diffusion zones produced by DRSE and the positive control (Clarithromycin, 1 µg/mL) (**B**), with the negative control (NC). (**C**) Crystal violet-stained microplate wells showing concentration-dependent inhibition of *H. pylori* biofilm formation at 25%, 50%, and 75% of the minimum bactericidal concentration.

The antibacterial and antibiofilm activities of DRSE were evaluated in comparison with the positive control (Table 6 and Figure 8). The control exhibited a significantly larger inhibition zone (25.00 ± 1.00 mm) than DRSE (19.70 ± 0.58 mm, *p* < 0.05), indicating stronger antibacterial activity. The minimum inhibitory concentration (MIC) of DRSE was 31.25 µg/mL, whereas the control displayed a lower MIC of 15.62 µg/mL, suggesting greater antimicrobial potency. Both treatments exhibited the same minimum bactericidal concentration (MBC) of 31.25 µg/mL; however, the MBC/MIC index was 1 for DRSE and 2 for the control. The antibiofilm assay demonstrated a concentration-dependent inhibition for both treatments. DRSE inhibited biofilm formation by 75.89 ± 2.50%, 87.77 ± 1.33%, and 95.39 ± 1.20% at 25, 50, and 75 µg/mL, respectively. The control exhibited significantly higher inhibition values of 86.34 ± 0.50%, 91.15 ± 2.50%, and 98.06 ± 0.50% at the corresponding concentrations (*p* < 0.05). As expected, the untreated control (0% MBC) showed no inhibition of biofilm formation, indicating maximal biofilm development in the absence of the test samples. Despite being less potent than the control, DRSE showed remarkable antibiofilm activity, achieving more than 95% inhibition at the highest tested concentration, highlighting its potential as a natural antibacterial and antibiofilm agent. Pure gallic acid produced inhibition zones of 8, 12, and 16 mm at concentrations of 250, 500, and 750 µg/mL, respectively, whereas vanillin produced inhibition zones of 8, 11, and 14 mm at the corresponding concentrations. Although both compounds exhibited measurable antibacterial activity against *H. pylori*, their inhibitory effects were observed only at relatively high concentrations. Moreover, even at the highest concentration tested (750 µg/mL), the inhibition zones produced by either compound remained smaller than those observed with crude DRSE, despite the extract being evaluated at a substantially lower concentration. These findings suggest that DRSE possesses greater antibacterial efficacy under the tested conditions. This enhanced activity may result from additive or synergistic interactions among the multiple bioactive phytochemicals present in the crude extract, rather than from the effects of gallic acid or vanillin alone.

Chhabra and Gupta [41] reported that *D. regia* flower extract exhibited noticeable antibacterial effects against *Shigella flexneri*, *Staphylococcus epidermidis*, and *Escherichia coli*, with inhibition zones ranging between 9 and 22 mm. These findings support the antimicrobial potential of *D. regia* seed constituents and highlight its activity against both Gram-positive and Gram-negative bacterial strains. Ghorpade et al. [42] reported that polysaccharides isolated from the ripe seed pulp of *D. regia* possess multiple gastrointestinal protective effects, including antioxidant activity and marked anti-ulcer properties, along with a favorable safety profile in acute toxicity testing. The extract was also shown to enhance gastric defense mechanisms and improve intestinal motility parameters in experimental models. These pharmacological effects suggest that *D. regia* seed-derived constituents may contribute to gastrointestinal protection, which may be relevant to its potential anti-*H. pylori* activity observed in seed extract-based studies. The anti-*H. pylori* activity observed in the present study may be linked to the diverse phytochemical constituents previously reported in *D. regia* seeds. Elhassaneen et al. [43] found that seed polysaccharides are enriched with phenolic compounds, saponins, tannins, carotenoids, and flavonoids and exhibit marked antioxidant activity. Since oxidative stress and gastric mucosal damage are closely associated with *H. pylori* infection, the antioxidant and protective properties of these seed constituents may contribute, at least in part, to the antimicrobial effects observed against this pathogen.

### 2.8. In Silico Characterization of Gallic Acid and Vanillin Interactions with Helicobacter pylori Urease

**Figure 9:**
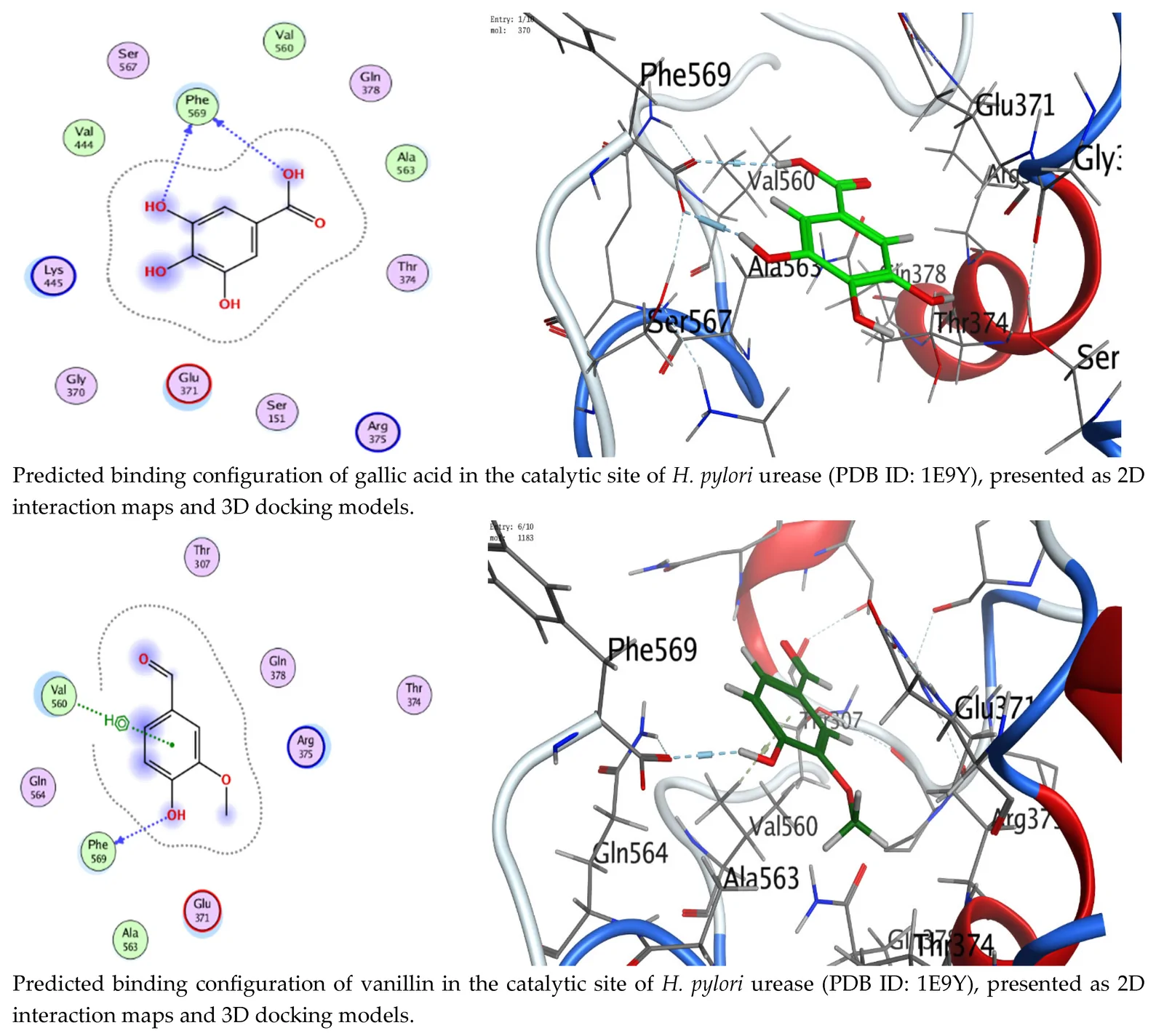
Predicted two- and three-dimensional binding modes of gallic acid and vanillin within the binding pocket of *Helicobacter pylori* urease (PDB ID: 1E9Y).

Molecular docking was used to investigate the predicted interactions of gallic acid, the most abundant detected phenolic, and vanillin, one of the least abundant detected constituents, with *H. pylori* urease (PDB ID: 1E9Y). The docking pocket was defined around the nickel-containing catalytic site, which comprises two nickel ions coordinated by His136, His138, His248, His274, Asp362, and carbamylated Lys219 (KCX219). Gallic acid and vanillin exhibited comparable top-ranked docking scores of −4.72 and −4.71 kcal/mol, respectively (Table 7 and Table 8). Gallic acid formed two hydrogen-bond donor interactions with Phe569 at distances of 2.93 and 3.43 Å. Vanillin formed one hydrogen bond with Phe569 and one π-H interaction with Val560. Phe569 and Val560 are positioned at the entrance to the active-site pocket, in close spatial proximity to key catalytic residues, including His274 and KCX219, and form part of the substrate-access channel. The corresponding two- and three-dimensional binding poses are documented (Figure 9). Urease is a central *H. pylori* virulence factor that generates ammonia from urea, buffers gastric acidity, and facilitates bacterial survival and colonization [44,45]. The predicted occupancy of the urease binding pocket therefore offers a plausible, although unconfirmed, molecular explanation for part of the observed antibacterial activity. The near-identical docking scores of gallic acid and vanillin despite their different concentrations illustrate that abundance in a crude extract does not necessarily predict target affinity. Instead, both major and minor metabolites may contribute through additive, synergistic, or complementary mechanisms. Phenolic compounds can also act through membrane disruption, metabolic enzyme inhibition, quorum-sensing interference, and attenuation of virulence pathways [46]. Gallic acid has previously shown direct antibacterial activity against *H. pylori* [47], supporting its possible contribution to the DRSE response. Nevertheless, docking predictions do not demonstrate enzyme inhibition; biochemical urease assays, isolated-compound testing, and molecular dynamics simulations are required to validate the proposed interactions. The comparable binding affinities of gallic acid and vanillin suggest that both abundant and minor DRSE constituents may contribute to the observed anti-*H. pylori* activity through complementary interactions with urease, providing a focused basis for subsequent enzymatic and molecular validation.

## 3. Materials and Methods

### 3.1. Plant Material Collection and Preparation of D. regia Seed Extract (DRSE)

Mature pods of *Delonix regia* (Bojer ex Hook.) Raf. were collected in April 2023, during the fruiting season, from healthy trees growing in Hail, Saudi Arabia. The collection and use of the plant material were conducted in accordance with the applicable institutional and national regulations of the Kingdom of Saudi Arabia, as well as relevant international guidelines governing plant research. The study also complied with the principles of the Convention on Biological Diversity (CBD) and the Convention on International Trade in Endangered Species of Wild Fauna and Flora (CITES). As *D. regia* is neither rare, threatened, nor endangered, no restrictions under these conventions applied to its collection or use in the present study. The plant material was identified on the basis of its morphological characteristics by a qualified botanist, Dr. Bazaid, at the Medical and Diagnostic Research Center (MDRC), University of Ha’il, Ha’il, Saudi Arabia. A voucher specimen was deposited in the herbarium of the Medical and Diagnostic Research Center under voucher specimen number UOH-MDRC-DR-2026-001. No genetic sequencing or molecular characterization was performed; therefore, no genetic data were generated or deposited in a public database, and no genetic accession numbers are associated with this study. Following collection, the pods were opened manually, and the seeds were separated, thoroughly washed with distilled water, and shade-dried at ambient temperature to a constant weight. The dried seeds were then ground into a fine powder using an electric laboratory mill and stored in airtight containers until extraction. Approximately 100 g of seed powder was immersed in 1 L of analytical-grade methanol at a solid-to-solvent ratio of 1:10 (*w*/*v*) and extracted by cold maceration for 72 h at ambient laboratory temperature with intermittent shaking. The extract was filtered through Whatman No. 1 filter paper, and the residual plant material was re-extracted twice under the same conditions to maximize the recovery of its constituents. The filtrates were pooled and concentrated under reduced pressure at 40 °C using a BÜCHI Rotavapor R-300 (BÜCHI Labortechnik AG, Flawil, Switzerland). The resulting concentrate was dried to a constant weight, designated as *D. regia* seed extract (DRSE), transferred to sterile amber-glass bottles, and stored at 4 °C until phytochemical characterization and biological evaluation. The extraction yield, calculated as the weight of the dried extract relative to the initial weight of the dry seed powder, was 12.5% (*w*/*w*).

### 3.2. High-Performance Liquid Chromatographic (HPLC) Analysis of Phenolic Compounds

The phenolic profile of DRSE was characterized using an Agilent 1260 Infinity HPLC system (Agilent Technologies, Santa Clara, CA, USA). Chromatographic separation was carried out on a Zorbax Eclipse Plus C8 analytical column (250 × 4.6 mm, 5 μm particle size). The mobile phase comprises water (solvent A) and acetonitrile supplemented with 0.05% trifluoroacetic acid (solvent B), with a constant flow rate of 0.9 mL min^−1^. A linear gradient elution program was employed as follows: 82% solvent A at the start of the run (0–1 min), gradually reduced to 75% from 1 to 11 min, followed by a further decrease to 60% between 11 and 18 min. The initial mobile-phase composition (82% A) was then restored from 18 to 22 min and maintained until the completion of the run at 24 min. The column compartment was thermostatically controlled at 40 °C, and chromatograms were monitored at 280 nm using a multi-wavelength detector. Each chromatographic analysis was performed with an injection volume of 5 μL. Instrument control, chromatographic acquisition, and data processing were conducted using Agilent ChemStation software (OpenLab ChemStation LTS 01.11). Individual phenolic compounds were identified by matching the retention times of sample peaks with those of authenticated reference standards analyzed under identical chromatographic conditions. Quantitative determination was achieved from the corresponding peak areas, and all integrations were manually inspected and verified to ensure reliable quantification [48,49]. The HPLC method was validated with respect to linearity, sensitivity, and precision. Calibration curves were constructed for each phenolic standard using six concentration levels over the range of 0.5–100 µg/mL. All calibration curves demonstrated good linearity, with coefficients of determination (R^2^) ranging from 0.995 to 0.999. The limits of detection (LOD) and quantification (LOQ) were determined at signal-to-noise ratios of 3:1 and 10:1, respectively. The LOD values ranged from 0.14 to 0.25 µg/mL, whereas the LOQ values ranged from 0.48 to 0.70 µg/mL. The regression equations, coefficients of determination, LOD values, and LOQ values for each compound are reported (Supplementary Table S1). All measurements were performed in triplicate, and the results are expressed as the mean ± SD.

### 3.3. Amino Acid Profiling

The amino acid composition of DRSE proteins was assessed according to the protocol described by Pellett and Young [50] with minor modifications. Briefly, 1 g of powdered sample was defatted using diethyl ether, and 0.4 g of the resulting material was incubated with 5 mL of 6 N HCl at 110 °C for 24 h. After hydrolysis, the solution was filtered through Whatman filter paper and evaporated to dryness in a water bath at 70 °C. The residue was reconstituted in a sample dilution solution, filtered through a 0.45 μm membrane filter, and stored in sealed vials at low temperature until analysis. Amino acid separation and quantification were performed using a Sykam amino acid analyzer (Sykam GmbH, Eresing, Germany) available at the Medical Diagnostic and Research Center (Hail, Saudi Arabia). Chromatographic analysis was carried out using a Sykam Hydrolysate column (LCA K06/Na), and amino acids were detected at dual wavelengths of 570 and 440 nm. The separation method employed was “AAA-Hydro” (Hydrolysate Separation), with an analysis time of 50 min and an injection volume of 0.1 mL. Data acquisition and peak integration were performed using Clarity Amino Acid Analyzer software (Version 10.2) (Clarity Amino Acid Analyzer SW, Sykam GmbH), and amino acid concentrations were calculated using external calibration based on the calibration file generated with authentic standards. Individual amino acids were identified according to their characteristic retention times and quantified from the corresponding peak areas.

### 3.4. Cytotoxicity Assay

The cytotoxic potential of DRSE was investigated against human epidermoid carcinoma (A431) and normal human fibroblast (HFB4) cell lines using the MTT colorimetric assay. Both cell lines were obtained from the American Type Culture Collection (ATCC, Manassas, VA, USA): A431 (ATCC^®^ CRL-1555^TM^) and HFB4 (ATCC^®^ CRL-2758^TM^). Both cell lines were maintained in Dulbecco’s Modified Eagle Medium (DMEM) (ThermoFisher Scientific, Waltham, MA, USA) supplemented with 10% fetal bovine serum (FBS), 100 U/mL penicillin, and 100 μg/mL streptomycin. Cell cultures were incubated at 37 °C under a humidified atmosphere containing 5% CO_2_. To assess the contribution of the predominant phenolic constituent to the overall anticancer activity of DRSE, the cytotoxicity of pure gallic acid was evaluated under the same experimental conditions used for the extract. Gallic acid (Sigma-Aldrich, St. Louis, MO, USA; purity ≥ 98%) was dissolved in dimethyl sulfoxide (DMSO) and serially diluted in culture medium to the required concentrations. The final concentration of DMSO in all treatment solutions did not exceed 0.1% (*v*/*v*). All experiments were conducted using cells at passage numbers below five. The cell cultures were routinely screened for mycoplasma contamination at monthly intervals using a PCR-based detection kit, and all tests yielded negative results. For the assay, cells were seeded into 96-well microplates at a density of 1 × 10^4^ cells per well and allowed to adhere for 24 h. The culture medium was then replaced with fresh medium containing DRSE at concentrations ranging from 31.25 to 1000 μg/mL, followed by incubation for 48 h. DRSE was dissolved in DMSO, and the final DMSO concentration in all treatment wells was maintained at ≤0.1% (*v*/*v*). Untreated cells maintained under identical conditions were used as the negative control. Cells treated with an equivalent volume of DMSO, at a final concentration of ≤0.1% (*v*/*v*), served as the vehicle control. Cisplatin (10 µM) and mitomycin C (5 µg/mL) were used as positive controls for A431 and HFB4 cells, respectively. After the treatment period, 20 μL of MTT solution (5 mg/mL) was added to each well, and the plates were incubated for an additional 4 h to permit intracellular reduction of MTT into insoluble formazan crystals. The culture medium was subsequently removed, and the formed crystals were solubilized in DMSO. Absorbance was measured at 570 nm using a microplate reader to determine cell viability. Each experiment was performed using triplicate wells as technical replicates and independently repeated three times as biological replicates. The half-maximal inhibitory concentration (IC_50_), defined as the concentration required to reduce cell viability by 50%, was determined from the dose–response curves by nonlinear regression using a four-parameter logistic model [log(inhibitor) versus normalized response with a variable slope] in GraphPad Prism version 8.0 (GraphPad Software, San Diego, CA, USA). IC_50_ values are expressed as the mean ± SD of three independent experiments. Moreover, cellular morphology was examined under an inverted phase-contrast microscope (Olympus CKX53, Olympus Corporation, Tokyo, Japan) at ×200 magnification. Representative images were captured using a digital camera attached to the microscope. Morphological alterations, including changes in cell density, cell shrinkage, membrane blebbing, rounding, and detachment from the culture surface, were recorded and compared with those of untreated control cells to evaluate the cytotoxic effects of DRSE [46,47]. The extent of cytotoxicity was calculated using the equation shown below.

$$\mathrm{Cytotoxicity}\;(\%)=\left(1-\frac{A_{t}}{A_{c}}\right)\times 100$$

where *A_t_* = absorbance of treated cells, *A_c_* = absorbance of untreated control cells.

### 3.5. In Vitro Scratch Wound-Healing Assay

The wound healing activity of DRSE was evaluated using an in vitro scratch wound healing assay on HFB4 cells. HFB4 cells were propagated in DMEM containing 10% fetal bovine serum (FBS), 100 U/mL penicillin, and 100 μg/mL streptomycin. Cultures were maintained under standard incubation conditions (37 °C, 5% CO_2_, humidified atmosphere). HFB4 cells were plated in 6-well culture plates and incubated until the cell monolayer reached approximately 90–100% confluence. A linear wound was then generated by gently scraping the monolayer with a sterile 200-μL pipette tip. Following scratch formation, the detached cells were carefully removed by washing the wells twice with phosphate-buffered saline (PBS). Fresh serum-free DMEM containing DRSE at a final concentration of 50 µg/mL was subsequently added to the treatment wells. This concentration was selected based on the cytotoxicity findings, which showed that HFB4 cell viability remained above 90%. Untreated cells served as the negative control, and cells exposed to an equivalent concentration of DMSO (≤0.1%, *v*/*v*) served as the vehicle control. The scratched regions were photographed at the time of wound creation (0 h) and after 48 h of incubation using an Olympus CKX53 inverted phase-contrast microscope (Olympus Corporation, Tokyo, Japan) equipped with a digital camera. Wound width (µm) and wound area (µm^2^) were quantitatively analyzed using ImageJ software (Version 1.54t) (National Institutes of Health, Bethesda, MD, USA) [51]. The wound healing response was evaluated by calculating the wound area difference, wound closure percentage, and cell migration rate according to the following equations.

$$\begin{array}{c} \mathrm{Area}\;\mathrm{difference}\;(µm^{2})=A_{0}-A_{48}\\ \mathrm{Wound}\;\mathrm{closure}\;(\%)=\frac{A_{0}-A_{48}}{A_{0}}\times 100\\ \mathrm{RM}=\frac{W_{0}-W_{48}}{t} \end{array}$$

where $A_{0}$ = wound area at 0 h (µm^2^), ${\;A}_{48}$ = wound area at 48 h (µm^2^), ${\;W}_{0}$ = wound width at 0 h (µm), $\;W_{48}$ = wound width at 48 h (µm),  t = incubation time (48 h).

### 3.6. Bovine Serum Albumin (BSA) Denaturation Assay for Anti-Inflammatory Activity

The anti-inflammatory potential of DRSE was evaluated using the BSA denaturation method with slight modifications [52]. Briefly, 50 µL of DRSE at different concentrations (1.56, 3.12, 6.25, 12.5, 25, 50, 100, and 200 µg/mL) was mixed with 450 µL of freshly prepared 1% (*w*/*v*) BSA solution. The reaction mixture was adjusted to pH 6.3 using 1 N HCl and gently mixed to ensure homogeneity. The reaction mixtures were initially kept at room temperature for 20 min to facilitate interaction between the extract and BSA. Subsequently, the mixtures were incubated in a thermostatically regulated water bath at 55 °C for 30 min to induce protein denaturation. After cooling to ambient temperature, the absorbance of each sample was measured at 670 nm using a BioSystem 310 Plus spectrophotometer (BioSystems S.A., Barcelona, Spain). Diclofenac sodium was included as the positive reference anti-inflammatory agent, while DMSO was used as the negative control. All measurements were conducted in triplicate to ensure reproducibility. The anti-inflammatory activity was expressed as the percentage inhibition of protein denaturation, calculated based on the equation presented below.

$$\mathrm{Inhibition}\;(\%)=\frac{A_{control}-A_{sample}}{A_{control}}\times 100$$

where $A_{control}$ = absorbance of the control, $A_{sample}$ = absorbance of the DRSE-treated sample.

### 3.7. In Vitro Anticoagulant Activity

The in vitro anticoagulant activity of DRSE was evaluated by measuring its effects on prothrombin time (PT) and activated partial thromboplastin time (APTT). Fresh human blood was collected from the corresponding author, Dr. Husam Qanash, following approval by the Ethics Committee of the University of Ha’il (reference no. H-2023-380; approval date: 16 October 2023). The blood was collected into tubes containing sodium citrate at a blood-to-anticoagulant ratio of 9:1 (*v*/*v*) for the preparation of citrated plasma. The plasma samples were separated by centrifugation and used immediately for coagulation analysis. DRSE was dissolved in sterile physiological saline (0.9% NaCl) and prepared at final concentrations of 25, 50, and 75 µg/mL. Heparin was employed as the reference anticoagulant, while untreated plasma served as the negative control. For each assay, an appropriate volume of plasma was mixed with the tested sample and incubated at 37 °C for 3 min before adding the corresponding coagulation reagent. For the PT assay, pre-warmed PT reagent was added to the plasma-sample mixture, and the clotting time was recorded automatically using a coagulation analyzer according to the manufacturer’s protocol. For the APTT assay, plasma was first incubated with APTT reagent at 37 °C; after that the addition of pre-warmed 0.025 M CaCl_2_ was added to initiate coagulation. The time required for clot formation was then recorded [53].

### 3.8. Anti-Helicobacter pylori Activity by Agar Well Diffusion Assay

The anti-*Helicobacter pylori* activity of the DRSE was evaluated using the agar well diffusion assay. The bacterial strain used in this study, *Helicobacter pylori* ATCC 43504, was obtained from the American Type Culture Collection (ATCC, Manassas, VA, USA). Fresh cultures of *H. pylori* were evenly inoculated onto the surface of Columbia blood agar plates supplemented with 5% defibrinated sheep blood using a sterile cotton swab to produce a uniform, confluent bacterial lawn. Sterile wells (6 mm in diameter) were then punched into the agar using a cork borer. A volume of 100 µL of each test sample at the desired concentration was carefully dispensed into the respective wells. Clarithromycin (1 µg/mL) was used as the positive control, while the corresponding solvent served as the negative control (DMSO at ≤0.1% *v*/*v*). The inoculated plates were left at room temperature for approximately 30 min to allow the test samples to diffuse into the agar. The plates were then incubated at 37 °C for 48-72 h under microaerophilic conditions (5% O_2_, 10% CO_2_, and 85% N_2_) in an anaerobic jar using a CampyGen gas-generating system (Oxoid, Basingstoke, UK). Following the incubation period, antibacterial activity was evaluated by measuring the diameters of the clear inhibition zones surrounding each well using a calibrated ruler, and the results were expressed in millimeters (mm) [54]. To assess the contribution of selected phytochemicals identified in DRSE to its anti-*H. pylori* activity, pure gallic acid and vanillin (Sigma-Aldrich, St. Louis, MO, USA; purity ≥ 98%) were individually evaluated for antibacterial activity at concentrations of 250, 500, and 750 µg/mL under the same experimental conditions used for the crude extract.

### 3.9. Minimum Bactericidal Concentration (MBC)

The broth microdilution assay was employed to determine the minimum inhibitory concentration (MIC) of the tested samples against *H. pylori* in sterile 96-well microplates. The assay was performed in accordance with the Clinical and Laboratory Standards Institute (CLSI) M07 guidelines, Methods for Dilution Antimicrobial Susceptibility Tests for Bacteria That Grow Aerobically. Serial two-fold dilutions were prepared to achieve a concentration range of 0.98–1000 µg/mL. Aliquots (200 µL) of the diluted samples were transferred into individual wells of the microplate. A fresh bacterial suspension was prepared in sterile 0.86% sodium chloride solution and adjusted to a turbidity equivalent to a McFarland standard of 1.1. Subsequently, 2 µL of the standardized inoculum was added to each well, resulting in a final bacterial density of approximately 5.0 × 10^4^ CFU/mL. Wells containing bacterial inoculum without the test sample served as the growth control, whereas wells containing the test sample without bacterial inoculation were included as the sterility control. The microplates were incubated at 37 °C for 72 h under microaerophilic conditions (5% O_2_, 10% CO_2_, and 85% N_2_). Following incubation, the minimum inhibitory concentration (MIC) was defined as the lowest concentration of the test sample that completely inhibited visible bacterial growth. To determine the minimum bactericidal concentration (MBC), 10 µL aliquots from wells showing no visible growth were aseptically inoculated onto Mueller-Hinton agar plates supplemented with 5% defibrinated sheep blood. The inoculated plates were incubated at 37 °C for 72 h, after which bacterial growth was examined. The MBC was recorded as the lowest concentration that produced no bacterial colonies on the agar surface, indicating complete bacterial killing. The ratio of MBC to MIC was subsequently calculated to characterize the antibacterial effect of the tested samples. A sample was considered to exhibit bactericidal activity when the MBC/MIC ratio was ≤4, whereas higher ratios were interpreted as indicative of a bacteriostatic effect [54].

### 3.10. Antibiofilm Activity Assay

The antibiofilm activity of the test samples against *H. pylori* ATCC 43504 was assessed by means of a crystal violet microtiter plate assay. Briefly, 250 µL of freshly prepared *H. pylori* suspension (1 × 10^6^ CFU/mL) in trypticase soy yeast broth was dispensed into each well of a sterile 96-well flat-bottom microplate. The bacterial suspension was supplemented with the test samples at sub-bactericidal concentrations corresponding to 25%, 50%, and 75% of the previously determined MBC. Clarithromycin (1 µg/mL) was included as the positive control for biofilm inhibition. Wells containing the bacterial inoculum without any test sample served as the untreated control (0% MBC), representing maximum biofilm formation and, consequently, 0% biofilm inhibition. The microplates were then incubated at 37 °C for 48 h under microaerophilic conditions (5% O_2_, 10% CO_2_, and 85% N_2_) to allow biofilm development. After incubation, the culture medium containing planktonic bacteria was carefully removed by aspiration, and the wells were gently rinsed with sterile distilled water to eliminate non-adherent bacterial cells. The plates were subsequently air-dried for approximately 35 min. Attached biofilms were stained using 0.1% crystal violet for 15–20 min at ambient temperature. Excess stain was eliminated by rinsing the wells with sterile distilled water, followed by complete air drying. The retained crystal violet was extracted with 250 µL of 95% ethanol, and optical density was determined at 575 nm after 20 min using a microplate reader [55]. Lower absorbance values indicated greater inhibition of *H. pylori* biofilm formation. All assays were performed in triplicate, and the results were expressed as the mean ± standard deviation.

### 3.11. Molecular Docking Study

Molecular docking simulations were conducted using the Molecular Operating Environment (MOE; Chemical Computing Group, Montreal, QC, Canada). The three-dimensional crystal structure of *H. pylori* urease (PDB ID: 1E9Y) was obtained from the Protein Data Bank (PDB). Before docking, water molecules and co-crystallized ligands were removed, while hydrogen atoms were added, and energy minimization was carried out using the default force field implemented in MOE.

Urease was selected as the molecular target because it is a major virulence factor that enables *H. pylori* to survive and colonize the acidic gastric environment by hydrolyzing urea into ammonia and carbon dioxide, thereby increasing the local pH. The enzyme is considered a promising therapeutic target because its catalytic pocket is highly conserved, which may reduce the likelihood of resistance arising through mutations at the target site. In addition, selective inhibition of bacterial urease may complement conventional antibiotic therapy while limiting disruption of the beneficial intestinal microbiota. The biological importance of urease, together with the availability of experimentally characterized inhibitor complexes, has therefore made it a widely investigated target in molecular docking studies.

The crystal structure of urease complexed with acetohydroxamic acid (AHA) was retrieved from the Protein Data Bank under PDB ID 1E9Y. The binding pocket was defined around the nickel-containing catalytic site, which represents the functional center of urease activity. This site contains two Ni^2+^ ions coordinated by His136, His138, His248, His274, Asp362, and carbamylated Lys219 (KCX219). Phe569 and Val560 are located at the entrance to the active-site pocket and form part of the substrate-access channel in close spatial proximity to the catalytic residues. This catalytic pocket was selected because it is directly responsible for enzymatic activity, is highly conserved, and contains the co-crystallized inhibitor AHA, which provides an experimentally validated reference for docking analysis. In the crystallographic structure, AHA coordinates with both nickel ions and forms hydrogen-bonding interactions with KCX219 and His274; therefore, its experimentally determined binding mode was used as a benchmark for evaluating the accuracy of the docking protocol.

The binding site was identified using the Site Finder module in MOE 2019, and its centroid was defined based on the coordinates of the co-crystallized AHA molecule and the two catalytic nickel ions. Dummy atoms were then generated to delineate the docking region and guide ligand placement within the catalytic pocket. The binding-pocket coordinates and key active-site residues are provided in Supplementary Table S2. The protein structure was subsequently prepared in MOE 2019, and protonation states were assigned using the software’s default settings at pH 7.0. The protonation states of the principal catalytic residues, particularly KCX219 and the active-site histidine residues, were manually inspected in accordance with their established roles in the urease catalytic mechanism. The two nickel ions were retained in their crystallographic positions and treated as rigid throughout the docking calculations, while their atomic coordinates and associated bond parameters were preserved from the original PDB structure. Potential ligand-metal coordination interactions were evaluated using the metal-coordination detection function implemented in MOE.

The chemical structures of gallic acid and vanillin were retrieved from the PubChem database using compound identifiers CID 370 and CID 1183, respectively. Both structures were subjected to geometry optimization before docking, and their conformations were generated using the conformational search algorithm implemented in MOE. Ligand poses were initially placed within the active site using the Triangle Matcher algorithm and scored using the London dG scoring function. The resulting poses were then refined through force-field-based energy minimization and rescored using the Generalized Born Volume Integral/Weighted Surface Area (GBVI/WSA dG) scoring function to estimate binding affinity. The top-ranked conformations were selected based on their docking scores and interaction patterns within the catalytic pocket. To validate the docking protocol, the co-crystallized AHA molecule was redocked into the active site, and its experimentally determined binding orientation, including coordination with both nickel ions and hydrogen-bonding interactions with KCX219 and His274, was used as the reference for assessing the ability of the docking procedure to reproduce the crystallographic pose and key active-site interactions.

### 3.12. Statistical Analysis

Data are presented as mean ± SD from three independent experiments (*n* = 3). Statistical analysis was performed using one-way ANOVA followed by Tukey’s post hoc test (*p* < 0.05). Different superscript letters indicate significant differences.

## 4. Conclusions

This study provides an integrated chemical, biological, and computational evaluation of *D. regia* seed extract, offering a broader understanding of its phytochemical composition and multifunctional biological activities. DRSE exhibited a phenolic profile rich in gallic acid, together with an amino acid fraction dominated by aspartic and glutamic acids. The extract showed selective cytotoxicity toward A431 epidermoid carcinoma cells, bactericidal and pronounced antibiofilm activity against *H. pylori*, substantial inhibition of protein denaturation, concentration-dependent anticoagulant activity, and a moderate stimulatory effect on fibroblast migration. The greater cytotoxic potency of DRSE compared with pure gallic acid suggests that its anticancer activity cannot be attributed to gallic acid alone, but may instead result from additive or synergistic interactions among the various bioactive phytochemicals present in the extract. Importantly, this study extends current knowledge of *D. regia* seeds by linking their detailed chemical composition to a range of biological activities and by providing molecular evidence for the potential interactions of both major and minor constituents with *H. pylori* urease. The comparable docking affinities of gallic acid and vanillin further support the possibility that the biological effects of DRSE arise from the complementary contributions of chemically diverse metabolites rather than from a single predominant compound. Overall, these findings identify DRSE as a promising natural source of multifunctional bioactive compounds and provide a scientific basis for further studies involving bioactivity-guided fractionation, mechanistic validation, comprehensive safety assessment, and in vivo evaluation.

## 5. Limitations and Future Perspectives

The findings of this study provide valuable preliminary evidence regarding the biological potential of DRSE; however, they should be interpreted within the scope of the experimental models used. The biological activities were evaluated mainly through in vitro assays, while the use of a crude extract reflects the combined effects of its diverse phytochemical constituents. In addition, the cytotoxicity assay focused on cell viability without examining specific mechanisms, such as apoptosis, cell-cycle regulation, or oxidative stress. The protein-denaturation and coagulation assays also served as useful screening approaches for evaluating anti-inflammatory and anticoagulant activity. Furthermore, the docking analysis provided initial molecular insights into the possible interactions of gallic acid and vanillin with *H. pylori* urease. Future investigations may build on these findings through bioassay-guided fractionation, isolated-compound and combination testing, mechanistic cellular studies, validated infection models, and appropriately designed in vivo experiments. Standardization of the extract, together with evaluation of its extraction yield, chemical stability, pharmacokinetic behavior, and safety profile, will further support assessment of its pharmaceutical potential.

## Figures and Tables

**Table 1:** Phenolic compounds and amino acid composition of *D. regia* seed extract.

Phenolic Compounds				Amino Acid		
Name	RT	Area	Conc. (µg/g)	Name	RT	Conc. (mg/g)
Gallic acid	3.55	547.90	1417.90	Aspartic acid	8.44	274.68
Chlorogenic acid	4.18	52.60	263.22	Threonine	10.30	17.62
Catechin	4.62	28.70	237.95	Serine	10.81	19.11
Methyl gallate	5.56	0.00	0.00	Glutamic acid	11.88	109.88
Caffeic acid	5.93	0.00	0.00	Proline	13.62	11.41
Syringic acid	6.26	9.56	21.86	Glycine	16.59	36.18
Rutin	6.62	7.87	40.86	Alanine	17.68	9.43
Ellagic acid	7.28	51.06	207.36	Cysteine	20.01	0.88
Coumaric acid	8.74	29.12	37.98	Valine	21.12	24.48
Vanillin	9.30	7.50	9.06	Methionine	23.30	0.77
Ferulic acid	9.73	29.29	60.49	Isoleucine	25.35	7.02
Naringenin	10.74	96.50	333.18	Leucine	26.55	24.54
Rosmarinic acid	12.04	89.50	284.01	Tyrosine	29.44	3.63
Daidzein	16.17	15.86	32.39	Phenylalanine	30.98	7.50
Quercetin	17.41	53.54	247.63	Histidine	35.86	5.81
Cinnamic acid	19.68	0.00	0.00	Lysine	36.77	15.38
Kaempferol	20.85	0.00	0.00	Ammonia	39.17	28.79
Hesperetin	21.52	0.00	0.00	Arginine	41.73	9.43

**Table 2:** Concentration-dependent cytotoxicity of *D. regia* seed extract in HFB4 fibroblasts and A431 epidermoid carcinoma cells.

Concentration (µg/mL)	Cytotoxicity (%)	
	HFB4 (Mean ± SD)	A431 (Mean ± SD)
0	0.00 ± 0.00 ^a^	0.00 ± 0.00 ^a^
31.25	0.00 ± 0.00 ^a^	0.09 ± 0.02 ^a^
62.5	0.11 ± 0.01 ^a^	30.23 ± 0.15 ^b^
125	2.42 ± 0.03 ^a^	55.23 ± 0.04 ^c^
250	30.07 ± 0.05 ^b^	85.75 ± 0.89 ^d^
500	96.94 ± 0.01 ^c^	97.19 ± 0.09 ^e^
1000	96.96 ± 0.04 ^c^	97.28 ± 0.96 ^e^
IC_50_	318.56 ± 2.06	108.97 ± 0.68

Data are presented as the mean ± SD of three independent experiments, each performed in triplicate. Within each cell line, different superscript letters indicate statistically significant differences among the tested concentrations (*p* < 0.05), as determined by one-way analysis of variance (ANOVA) followed by Tukey’s post hoc test. Values sharing the same superscript letter are not significantly different. IC_50_ values were calculated by nonlinear regression using a four-parameter logistic model in GraphPad Prism version 8.0. Cisplatin (10 µM) and mitomycin C (5 µg/mL) were used as positive controls for the A431 and HFB4 cell lines, respectively.

**Table 3:** Quantitative evaluation of the wound-healing activity of *D. regia* seed extract (DRSE) using the in vitro scratch assay.

Treatment	At 0 h		At 48 h		RM (µm)	Wound Closure (%)	Area Difference (µm^2^)
	Mean Width (µm)	Mean Area (µm^2^)	Mean Width (µm)	Mean Area (µm^2^)			
Control	844.67	908,618.41	161.70	174,018.5	14.23 ^a^	80.85 ^a^	734,599.9 ^a^
DRSE	946.68	1,018,351.46	173.07	186,214.1	16.12 ^b^	81.71 ^a^	832,137.4 ^b^

DRSE was evaluated at a concentration of 50 µg/mL. Data are presented as the mean of three independent experiments. For each parameter, different superscript letters (a and b) indicate a statistically significant difference between the control and DRSE-treated groups (*p* < 0.05), as determined using an unpaired *t*-test, whereas values sharing the same superscript letter indicate no statistically significant difference. RM, migration rate.

**Table 4:** Inhibition of bovine serum albumin (BSA) denaturation by *D. regia* seed extract (DRSE) and diclofenac sodium.

Concentration (µg/mL)	Anti-Inflammatory (%)	
	Diclofenac Sodium (%)	DRSE (%)
0.0	0.00 ± 0.00 ^a^	0.00 ± 0.00 ^a^
1.56	47.00 ± 0.87 ^a^	32.50 ± 0.50 ^b^
3.12	56.50 ± 0.50 ^a^	41.70 ± 0.20 ^b^
6.25	64.80 ± 0.17 ^a^	49.20 ± 0.20 ^b^
12.5	74.00 ± 0.87 ^a^	58.67 ± 0.29 ^b^
25	82.40 ± 0.40 ^a^	66.70 ± 0.20 ^b^
50	90.50 ± 0.44 ^a^	76.00 ± 0.80 ^b^
100	94.70 ± 0.10 ^a^	85.10 ± 0.10 ^b^
200	98.30 ± 0.89 ^a^	91.20 ± 0.26 ^b^
IC_50_	1.63 ± 0.8	6.39 ± 0.19

Data are presented as the mean ± SD of three independent experiments. At each concentration, different superscript letters (a and b) indicate statistically significant differences between DRSE and diclofenac sodium (*p* < 0.05), as determined by one-way analysis of variance (ANOVA) followed by Tukey’s post hoc test. Values sharing the same superscript letter indicate no statistically significant difference between the two treatments at the corresponding concentration. IC_50_ values represent the concentration required to achieve 50% inhibition and were determined by nonlinear regression analysis using GraphPad Prism version 8.0. IC_50_ values are presented as the mean ± SD.

**Table 5:** Effects of *D. regia* seed extract (DRSE) and heparin on prothrombin time (PT) and partial thromboplastin time (PTT).

Concentration (µg/mL)	PT		PTT	
	DRSE	Heparin	DRSE	Heparin
0	12.00 ± 1.00 ^a^	12.00 ± 1.00 ^a^	25.00 ± 1.00 ^a^	25.00 ± 1.00 ^a^
25	12.17 ± 0.29 ^b^	22.33 ± 0.29 ^a^	23.50 ± 0.50 ^b^	76.67 ± 0.58 ^a^
50	14.70 ± 0.17 ^b^	154.00 ± 1.73 ^a^	50.80 ± 0.20 ^b^	215.00 ± 3.00 ^a^
75	27.37 ± 0.15 ^b^	210.67 ± 0.58 ^a^	90.50 ± 0.50 ^b^	324.00 ± 1.00 ^a^

Data are presented as the mean ± SD of three independent experiments. At each concentration, different superscript letters (a and b) indicate a statistically significant difference between DRSE and heparin (*p* < 0.05), as determined using an unpaired *t*-test, whereas values sharing the same superscript letter indicate no statistically significant difference between the two treatments. PT, prothrombin time; PTT, partial thromboplastin time.

**Table 6:** Antibacterial, bactericidal and antibiofilm activities of *D. regia* seed extract (DRSE) against *Helicobacter pylori*.

Treatment	IZ (mm)	MIC (µg/mL)	MBC (µg/mL)	MBC/MIC Index	Antibiofilm Activity % at Different MBC (%)			
					** 0.0	25	50	75
DRSE	19.7 ± 0.58 ^a^	31.25 ^a^	31.25 ^a^	1	0.0	75.89 ± 2.5 ^a^	87.77 ± 1.33 ^a^	95.39 ± 1.2 ^a^
* Control	25.0 ± 1.0 ^b^	15.62 ^b^	31.25 ^a^	2	0.0	86.34 ± 0.5 ^b^	91.15 ± 2.5 ^b^	98.06 ± 0.5 ^b^

Data are presented as the mean ± standard deviation. Within each column, different superscript letters (a and b) indicate statistically significant differences among treatments (*p* < 0.05), whereas values sharing the same superscript letter are not significantly different. Statistical analysis was performed using one-way analysis of variance followed by Tukey’s multiple-comparison test. * Clarithromycin (1 µg/mL) was used as the positive control in the antibacterial and antibiofilm assays. ** The value of 0.0% represents the untreated control, which contained the bacterial inoculum without any test sample and corresponds to 0% biofilm inhibition.

**Table 7:** Docking scores and energy terms for gallic acid and vanillin with *Helicobacter pylori* urease (PDB ID: 1E9Y).

Mol	S	Rmsd_Refine	E_Conf	E_Place	E_Score1	E_Refine	E_Score2
Gallic acid	−4.71924	0.88683438	−32.7976	−61.0947	−11.5713	−23.6458	−4.71924
Gallic acid	−4.63386	1.0563314	−32.6535	−65.6089	−10.6118	−21.8702	−4.63386
Gallic acid	−4.5831	0.92853487	−33.0623	−54.9958	−10.7385	−21.945	−4.5831
Gallic acid	−4.56335	0.67297304	−32.8618	−60.0124	−10.7821	−19.3995	−4.56335
Gallic acid	−4.55105	1.1615303	−32.7204	−54.7859	−12.3389	−20.1944	−4.55105
Vanillin	−4.71045	0.95530742	−8.8851	−57.9966	−8.9113	−20.9196	−4.71045
Vanillin	−4.6975	0.89590669	−8.95271	−60.2181	−8.29444	−21.7954	−4.6975
Vanillin	−4.57006	1.0638454	−9.11366	−59.548	−8.32263	−20.3978	−4.57006
Vanillin	−4.55137	1.2195598	−7.20216	−55.3423	−8.71471	−20.9035	−4.55137
Vanillin	−4.54543	0.93095404	−9.95616	−67.3923	−8.60585	−17.8374	−4.54543

**Table 8:** Predicted molecular interactions of gallic acid and vanillin with *Helicobacter pylori* urease (PDB ID: 1E9Y).

Mol	Ligand	Receptor	Interaction	Distance	E (kcal/mol)
Gallic Acid	O 3	OXT PHE 569 (B)	H-donor	2.93	−3.7
	O 4	O PHE 569 (B)	H-donor	3.43	−1.0
Vanillin	O 2	O PHE 569 (B)	H-donor	3.04	−2.0
	6-ring	CG2 VAL 560 (B)	pi-H	3.68	−0.7

## Data Availability

Data that supports the findings of this study are available within the article and from the corresponding author upon request.

## Supplementary Materials

The following supporting information can be downloaded at: https://www.mdpi.com/article/10.3390/ph19081272/s1, Table S1: Validation parameters for the HPLC analysis of phenolic compounds in *D. regia* seed extract (DRSE); Table S2: Active-site pocket coordinates and key residues of *Helicobacter pylori* urease (PDB ID: 1E9Y) used for molecular docking.
