# Supplementary material for: Natural Bioactive Compounds from Delonix regia Seeds Revealed Through Integrated Phytochemical, Biomedical and In Silico Evaluation
Source: Pharmaceuticals (Basel). 2026 Aug 12;19(8):1272. doi: 10.3390/ph19081272 (PMC13516390; doi:10.3390/ph19081272)
Supplement: Supplementary file 1 [file pharmaceuticals-19-01272-s001.zip › pharmaceuticals-4475663-supplementary.pdf]

Natural Bioactive Compounds from *Delonix regia* Seeds Revealed through Integrated Phytochemical, Biomedical and *In Silico* Evaluation

Husam Qanash [1,2,*], Aisha M. H. Al-Rajhi [3], Abdulrahman S. Bazaid [1,2], Manar F. Alghassab [2,4], Fahad Almarshadi [5], Walid Alesefir [6], Waleed Hakami [7], Amro Duhduh [7] and Abdu Aldarhami [8]

[1] Department of Medical Laboratory Science, College of Applied Medical Sciences, University of Ha'il, Hail 55476, Saudi Arabia
[2] Medical and Diagnostic Research Center, University of Ha'il, Hail 55473, Saudi Arabia
[3] Department of Biology, College of Science, Princess Nourah bint Abdulrahman University, P.O. Box 84428, Riyadh 11671, Saudi Arabia
[4] College of Dentistry, University of Ha'il, Hail 55473, Saudi Arabia
[5] Public Health Department, College of Public Health and Health Informatics, University of Ha'il, Hail 55473, Saudi Arabia
[6] Department of Internal Medicine, College of Medicine, University of Ha'il, Hail 55473, Saudi Arabia
[7] Department of Medical Laboratory Technology, College of Nursing and Health Sciences, Jazan University, Jazan 45142, Saudi Arabia
[8] Department of Microbiology and Parasitology, Faculty of Medicine, Umm Al-Qura University, Al-Qunfudah 28815, Saudi Arabia
* Correspondence: h.qanash@uoh.edu.sa

Supplementary Table S1. Validation parameters for the HPLC analysis of phenolic compounds in *D. regia* seed extract (DRSE).

Compound	RT (min)	Calibration Range (μg/mL)	Regression Equation	R^2	LOD (μg/mL)	LOQ (μg/mL)	Conc. (μg/g)
**Gallic acid**	3.55	0.5–100	y = 45231x + 125.6	0.999	0.15	0.50	1417.90
**Chlorogenic acid**	4.18	0.5–100	y = 38762x + 98.3	0.998	0.18	0.55	263.22
**Catechin**	4.62	0.5–100	y = 31245x + 87.4	0.997	0.20	0.60	237.95
**Syringic acid**	6.26	0.5–100	y = 28451x + 76.2	0.996	0.22	0.65	21.86
**Rutin**	6.62	0.5–100	y = 36587x + 94.1	0.998	0.16	0.52	40.86
**Ellagic acid**	7.28	0.5–100	y = 33412x + 88.5	0.997	0.19	0.58	207.36
**p-Coumaric acid**	8.74	0.5–100	y = 29563x + 79.8	0.996	0.21	0.63	37.98
**Vanillin**	9.30	0.5–100	y = 27438x + 72.3	0.995	0.25	0.70	9.06
**Ferulic acid**	9.73	0.5–100	y = 35621x + 91.6	0.998	0.17	0.53	60.49
**Naringenin**	10.74	0.5–100	y = 37895x + 96.7	0.997	0.18	0.56	333.18
**Rosmarinic acid**	12.04	0.5–100	y = 41876x + 108.4	0.999	0.14	0.48	284.01
**Daidzein**	16.17	0.5–100	y = 28976x + 78.1	0.996	0.23	0.68	32.39
**Quercetin**	17.41	0.5–100	y = 37123x + 95.2	0.998	0.16	0.51	247.63

RT, retention time; LOD, limit of detection; LOQ, limit of quantification; R^2, coefficient of determination; Conc., concentration. Note**:** Methyl gallate, caffeic acid, cinnamic acid, kaempferol, and hesperetin were not detected under the chromatographic conditions used and were therefore excluded from the table.

Supplementary Table S2. Active-site pocket coordinates and key residues of *Helicobacter pylori* urease (PDB ID: 1E9Y) used for molecular docking.

Parameter	Details
**PDB ID**	1E9Y
**Resolution**	3.00 Å
**Active site centroid (x, y, z)**	[Insert coordinates, e.g., -12.34, 25.67, 8.90]
**Pocket radius**	8.0 Å
**Nickel ions**	Ni^1 (chain B, residue 3001), Ni^2 (chain B, residue 3002)
**Key catalytic residues**	His136, His138, His248, His274, Asp362, KCX219 (carbamylated Lys219)
**Co-crystallized ligand**	Acetohydroxamic acid (AHA)
**Redocking RMSD**	1.42 Å

Pocket centroid coordinates are based on the geometric center of the co-crystallized ligand AHA and the two nickel ions.
